# Mocravimod as a repurposing drug against clinical isolates of *Staphylococcus aureus* by targeting cell membrane

**DOI:** 10.1128/spectrum.02263-25

**Published:** 2026-07-27

**Authors:** Yuanyuan Tang, Yuqing Xia, Xuancheng Huang, Zhijian Yu, Duoyun Li, Shiqing Han, Peiyu Li, Tieying Hou

**Affiliations:** 1 College of Biotechnology and Pharmaceutical Engineering, Nanjing Tech University 626877, Nanjing, China; 2 Department of Infectious Diseases and Shenzhen Key Laboratory for Endogenous Infections, Shenzhen Nanshan People’s Hospital, Affiliated Nanshan Hospital of Shenzhen University 194030 , Shenzhen, China; 3 School of Pharmacy, Shenzhen University Medical School, Shenzhen University 481870, Shenzhen, China; Shenzhen University School of Medicine, Shenzhen, China; West Virginia University, Morgantown, West Virginia, USA

**Keywords:** mocravimod hydrochloride, *Staphylococcus aureus*, biofilms, membrane integrity, antibacterial activity, sphingosine-1-phosphate receptor modulator

## Abstract

*Staphylococcus aureus* infections, particularly those caused by multidrug-resistant strains and associated with biofilm formation, pose a major therapeutic challenge in clinical practice. The objective of this study was to evaluate the antibacterial and antibiofilm activity of mocravimod (KRP-203), an FDA-approved S1P receptor modulator, against clinical *S. aureus* isolates and to explore its underlying mechanism of action. The antibacterial activity of KRP-203 was assessed against methicillin-susceptible *S. aureus* (MSSA) and methicillin-resistant *S. aureus* (MRSA) using MIC determination, time-kill assays, and biofilm inhibition models. KRP-203 exhibited strong bactericidal activity against planktonic MSSA and MRSA, with MIC values ranging 6.25–50μM. Time-kill assays demonstrated rapid bacterial eradication at 8× MIC within 2 h, showing superior killing kinetics compared with vancomycin. At sub-inhibitory concentrations, KRP-203 inhibited biofilm formation by up to 70% and reduced viable bacterial counts in mature biofilms by >2.5 logs. To elucidate the antibacterial mechanism, whole-genome sequencing and quantitative proteomic analyses were performed. These analyses revealed mutations in membrane-associated genes, including glnQ and BCAT, and significant alterations in proteins related to membrane integrity and redox regulation. Consistently, functional assays confirmed that KRP-203 disrupts bacterial cell membrane, as evidenced by dose-dependent membrane depolarization, increased permeability, and direct binding to cardiolipin and phosphatidylglycerol. Molecular docking further predicted a favorable interaction between KRP-203 and GlnQ. In conclusion, KRP-203 demonstrated notable antibacterial and antibiofilm activity against *S. aureus*, likely through membrane integrity disruption. While these findings highlight its potential as a repurposed antibacterial agent, further studies are required to fully elucidate its molecular targets, optimize antibacterial efficacy, and evaluate its *in vivo* safety profile.

**IMPORTANCE:**

Antibiotic resistance and the formation of biofilms, which protect bacteria from medications and immunological responses, present the significant challenges for the clinical treatment of *Staphylococcus aureus* infections. This study reveals mocravimod hydrochloride (KRP-203), a clinically approved drug initially intended to treat leukemia, as a viable new candidate against S. aureus infection. KRP-203 quickly kills both drug-susceptible and resistant *S. aureus*, including difficult-to-treat biofilm-associated cells. Its membrane-disrupting activity quickly kills drug-resistant bacteria while also destroying biofilm formations, presenting a dual action rarely accomplished by conventional antibiotics. Critically, KRP-203’s established safety profile in human studies may hasten its repurposing as a new weapon against biofilm-associated infections, providing possible solutions for chronic and drug-resistant *S. aureus* infections where existing treatments commonly fail.

## INTRODUCTION

*Staphylococcus aureus* is still one of the most common and clinically relevant bacteria involved in both hospital-acquired and community-associated infections, ranging from superficial skin lesions to life-threatening diseases such as pneumonia, osteomyelitis, and sepsis (1, 2). The widespread emergence of antibiotic-resistant strains, such as methicillin-resistant *S. aureus* (MRSA), vancomycin-intermediate *S. aureus* (VISA), and strains resistant to linezolid and daptomycin, has made the therapeutic management of *S. aureus* infections more challenging in recent decades (3). According to the 2024 report from CHINET (https://www.chinets.com/Data/AntibioticDrugFast), the detection rate of MRSA in clinical *S. aureus* isolates has shown a gradual decline since 2005, reaching 29.2% in 2024. Nevertheless, *S. aureus* continues to rank among the top five most frequently isolated bacterial pathogens. Surveillance statistics from China and other countries reveal an alarming number of resistance isolates, underscoring the critical need for innovative strategies to combat these persistent illnesses (4). The limited efficacy of current antimicrobial medicines against these multidrug-resistant (MDR) infections has drastically reduced treatment options and contributed to global morbidity and mortality increases (5).

Beyond traditional resistance mechanisms, *S. aureus* has a remarkable ability to persist and elude treatment clearance by forming biofilms—a highly organized colony of bacterial cells enclosed in a self-produced extracellular matrix (6). These biofilm structures often cling to biotic and abiotic surfaces, including implanted medical devices, and act as a protective niche, limiting drug penetration while shielding resident bacteria from host immune responses (7). As a result, biofilm-related infections are notoriously difficult to cure, frequently necessitating lengthy antibiotic rounds or surgical intervention (8). Furthermore, biofilm-associated cells may include dormant persister populations, complicating eradication and leading to infection recurrence (9). The twin issue of resistance and biofilm-mediated tolerance has rendered many traditional antibiotics useless in clinical settings, particularly against chronic or device-associated illnesses (10, 11).

**Fig 1:**
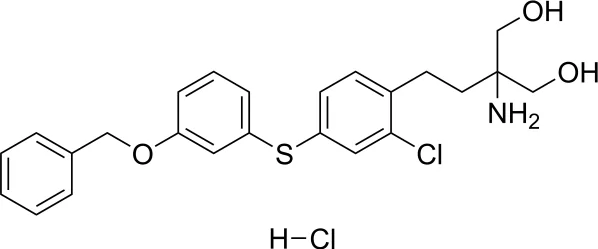
Chemical structure of mocravimod hydrochloride.

Given these obstacles, finding new medicines capable of targeting both planktonic and biofilm-embedded bacterial cells is critical (12). Drug repurposing, or reevaluating existing compounds for new antimicrobial activity, has emerged as a practical and effective way to accelerate anti-infective drug discovery by skipping the early stages of pharmacokinetic and safety testing (13, 14). Mocravimod hydrochloride (KRP-203) is a synthetic sphingosine-1-phosphate receptor (S1PR) modulator that is phosphorylated *in vivo* to its active form, mocravimod-phosphate, which targets four of the five S1PR subtypes with high selectivity for S1P1 (15). Through modulation of S1PR signaling, KRP-203 regulates lymphocyte trafficking by preventing T-cell egress from lymphoid organs while preserving cytotoxic T-cell function (16). The chemical structure of KRP-203 is shown in Fig. 1. It is currently under investigation in a Phase 3 clinical trial (ClinicalTrials. gov identifier NCT05429632) for the treatment of adult acute myeloid leukemia. The antibacterial activity of KRP-203 was initially discovered by scanning a specific library of FDA-approved clinical medicines. While KRP-203 has shown excellent safety profiles in preclinical models for different applications (15, 17), its antibacterial potential has not yet been investigated. Thus, the goal of this study was to assess KRP-203’s antibacterial and anti-biofilm capabilities against *S. aureus*. The possible antibacterial mechanism was investigated further by inducing KRP-203-resistant *S. aureus* strains *in vitro* and then sequencing their entire genome. Additionally, a label-free global proteome analysis was performed to investigate the effect of KRP-203 on bacterial protein expression and its potential antibacterial mechanisms. Through hypothesis and a series of verifications, KRP-203’s disruption of bacterial cell membrane integrity was proved as a possible mode of action. The purpose of this study is to provide new insights into KRP-203’s repurposing potential as a dual-function therapeutic agent for treating drug-resistant and biofilm-associated *S. aureus* infections.

## RESULTS

### KRP-203 exhibits superior bactericidal activity against gram-positive bacteria

**Fig 2:**
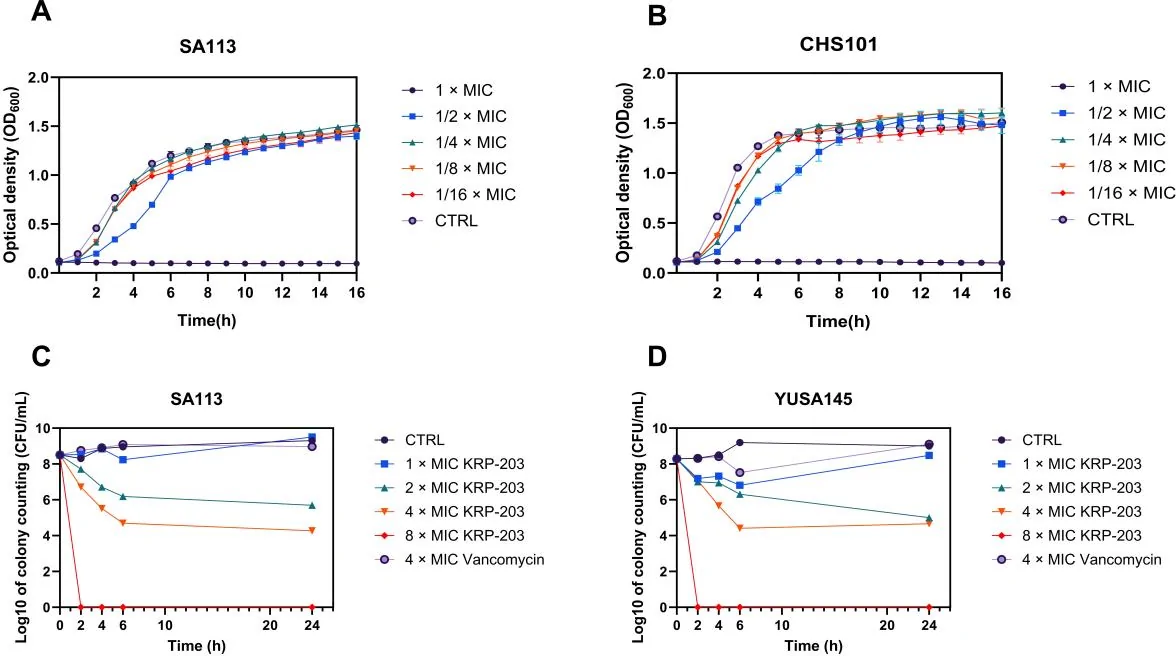
KRP-203 significantly inhibited the growth of *S. aureus*. (**A and B**) The planktonic cells of MSSA SA113 and CHS101 were treated with 1/16 ×, 1/8 ×, 1/4 ×, 1/2 ×, and 1× MIC of KRP-203. The OD_600_ value of bacterial cells was measured by bioscreen C (Turku, Finland) at 1-h intervals for 16 h. TSB without antimicrobial was used as a negative control. Data are shown as Mean ± SEM (*n* = 3). All experiments were performed twice. (**C**) *S*. *aureus* SA113 and (**D**) YUSA145 were challenged with 1×, 2×, 4×, and 8× MIC of KRP-203 as well as 4× MIC of vancomycin. Bacterial growth in TSB without antimicrobial was used as a negative control. Data are representative of three independent experiments.

In order to evaluate the antibacterial activity of KRP-203, the MICs of KRP-203 were determined in 21 clinical *E. faecalis* strains, 19 clinical *E. faecium* strains, 22 clinical *S. epidermidis* strains, and 39 clinical *S. aureus* strains from China, including 19 methicillin-susceptible *S. aureus* (MSSA) isolates and 20 MRSA isolates. KRP-203’s MIC ranged from 6.25 μM (≈3.0 μg/mL) to 50 μM (≈24 μg/mL, Table 1). It is particularly effective against *E. faecalis*, with a MIC_90_ of only 12.5 μM, and the particular MIC values for various bacteria are available in the supplemental materials (Table S1). Besides, KRP-203 inhibited the planktonic growth of *S. aureus*, as demonstrated by the bacteria automatic growth curve experiment. A concentration of 1× MIC completely inhibited the planktonic growth of MSSA SA113 and CHS101 (Fig. 2A and B). It should be noted that at a dose of 1/2× MIC, KRP-203 inhibited planktonic growth of SA113 and CHS101 but did not affect bacterial growth after 16 h.

**TABLE 1:** MIC distribution of KRP-203 against gram-positive bacterial isolates^*a*^

Bacteria species	KRP-203 MIC values (μM)				MIC_50_/MIC_90_
	6.25	12.5	25	50	
*MRSA* (*n* = 20)	0	4	9	7	25/50
*MSSA* (*n* = 19)	0	6	6	7	25/50
*S. epidermidis* (*n* = 22)	0	18	4	0	12.5/25
*E. faecium* (*n* = 19)	0	19	0	0	12.5/12.5
*E. faecalis* (*n* = 21)	2	3	16	0	25/25

^*a*^ MIC50/MIC90, the MIC values for 50% or 90% of bacterial growth inhibition.

To explore KRP-203’s antibacterial efficacy in a dose-dependent manner, a time-kill experiment against *S. aureus* was performed in comparison with vancomycin, a traditional bactericide. As indicated in Fig. 2C and D, the time-kill curve experiment revealed that KRP-203’s bactericidal activity in SA113 and YUSA145 was better than 4× MIC of vancomycin. In addition, KRP-203 with 8× MIC had a robust eradication effect on SA113 and YUSA145 at 2 h after drug administration, indicating its quick and efficient bactericidal activity against planktonic bacteria.

### Antibiofilm activity of KRP-203

**Fig 3:**
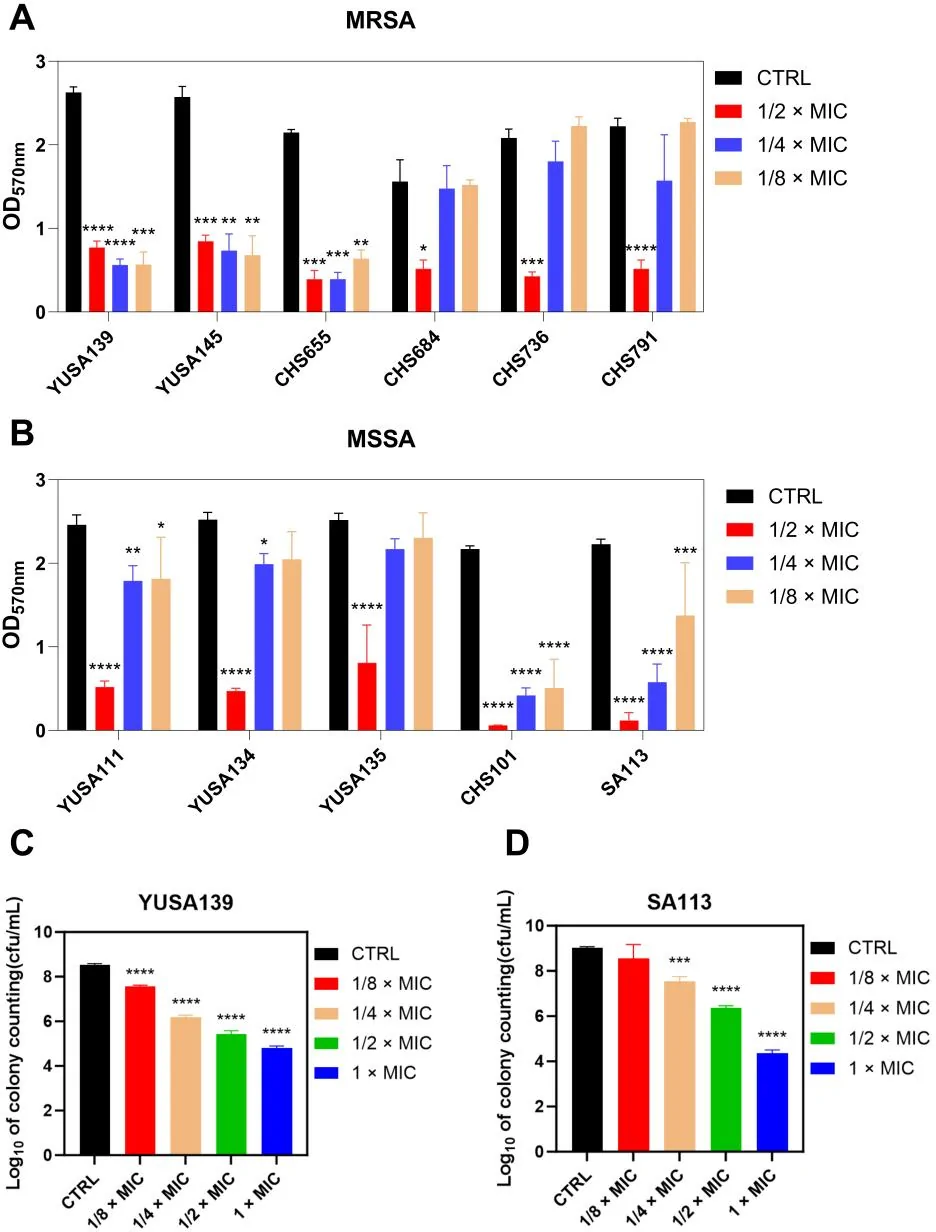
Antibiofilm activity of KRP-203 against *S. aureus*. (**A**) MRSA and (**B**) MSSA isolates were tested for the inhibitory effect of KRP-203 on the biofilm formation under different concentrations. The reduction in CFU count of *S. aureus* (**C**) YUSA139 and (**D**) SA113 cells in biofilms at various concentrations of KRP-203 was compared with the corresponding untreated biofilms. Data are represented as means ± SD, *N* = 3. *P < 0.05; **P < 0.01 (two-tailed Student’s *t*-test).

The effect of KRP-203 on *S. aureus* biofilm formation was investigated using *S. aureus* clinical isolates, including MSSA and MRSA, by the method of crystal violet staining. In Fig. 3A and B, KRP-203’s anti-biofilm activity at sub-MIC levels (1/8 ×, 1/4 ×, and 1/2 × MIC) significantly inhibited *S. aureus* biofilm formation in a dose-dependent manner, particularly at 1/2× MIC. Concurrently, in the majority of *S. aureus* clinical isolates, biofilm formation was reduced by roughly 70%, demonstrating that AZD-5991 could significantly inhibit *S. aureus* biofilm formation. Moreover, KRP-203 treatment (1/2× MIC) reduced bacteria entrenched in mature biofilms of *S. aureus* YUSA139 by over 3 logs and 2.5 logs of SA113, as seen in Fig. 3C and D. These findings suggest that KRP-203 is highly effective against the biofilm formation of *S. aureus* once again.

### Genetic mutation in KRP-203-induced *S. aureus*

**Fig 4:**
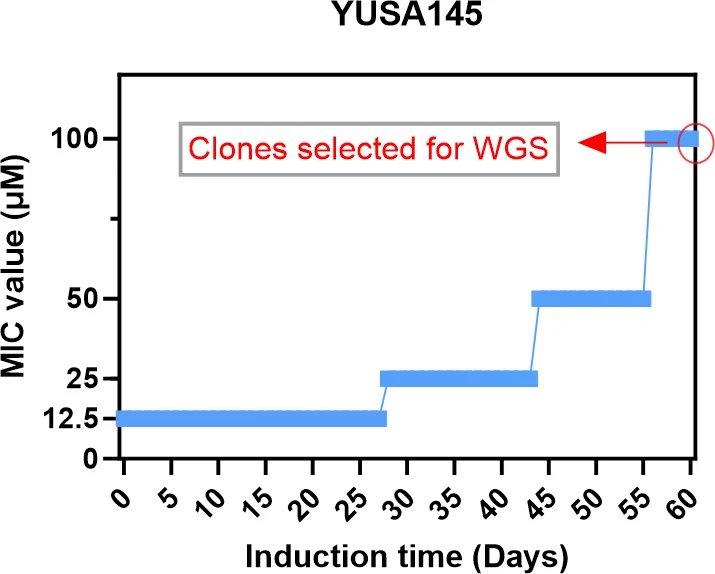
Dynamics of KRP-203 MIC values in KRP-203-induced *S. aureus* YUSA145. The *S. aureus* YUSA145 isolate was serially subcultured in TSB with KRP-203, starting with an inducing concentration of 1/2× MIC and gradually increasing to higher concentrations. WGS: whole-genome sequencing.

After 60 generations of continuous passages of YUSA145 under KRP-203 pressure *in vitro*, the KRP-203-induced resistant *S. aureus*, designated as YUSA145N60, was selected, and its MIC of KRP-203 was enhanced by eightfold from 12.5 to 100 μM (Fig. 4). Then, whole-genome sequencing was used to identify genetic mutations in the KRP-203-induced tolerant *S. aureus* strain YUSA145N60. Three nonsynonymous mutations were found in three functional genes: recombination factor protein RarA, glutamine transport ATP-binding protein glnQ, and branched-chain amino acid aminotransferase (Table 2). Among them, glnQ, the ATP-binding component of the ABC transporter complex, hydrolyzes ATP in the cytoplasm and couples to its membrane-integrated partner (e.g., GlnP) to actively translocate glutamine across the cytoplasmic membrane. Moreover, branched-chain amino acid aminotransferase (BCAT) participates in the metabolism of branched-chain amino acids (BCAAs), generating metabolites that serve as precursors for membrane lipid biosynthesis, and is thus intimately linked to bacterial membrane phospholipid formation. As a result, it was suggested that KRP-203 exposure could affect phospholipid formation pathway and cell membrane production.

**TABLE 2:** Determination of genetic mutations in *S. aureus* YUSA145 by whole-genome sequencing

Ref_gene_ID	Mutation type	NC mutations	AA mutations	Subject description
YUSA145_GM001612	nonsyn	C172T	R58C	Recombination factor protein RarA
YUSA145_GM001896	nonsyn	C754T	H252Y	Glutamine transport ATP-binding protein glnQ
YUSA145_GM001974	nonsyn	C746T	A249V	Branched-chain amino acid aminotransferase

### Proteomic analysis of *S. aureus* treated with KRP-203

**Fig 5:**
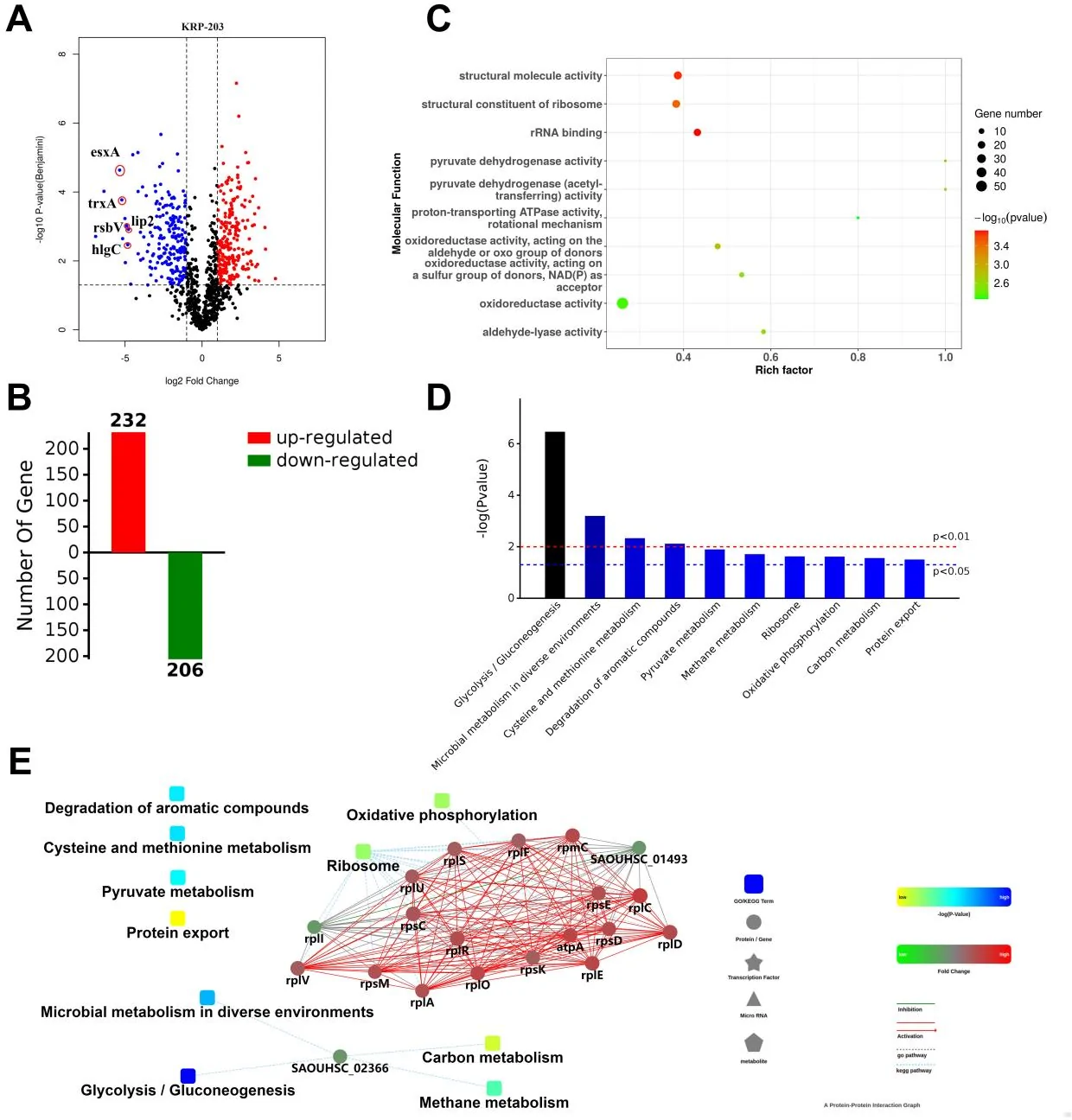
Proteomic response of *S. aureus* SA113 to KRP-203. (**A and B**) Differential protein volcano plot and statistical analysis after treatment with a sub-inhibitory concentration of KRP-203. The *x*-axis represents the fold change of differentially expressed proteins between the KRP-203 treated group and the untreated group of *S. aureus*, and the *y*-axis represents the significance of the difference (*P*-value). Red dots represent upregulated proteins after treatment, while blue dots represent downregulated proteins. (**C**) Gene Ontology (GO) biological process analysis of differentially expressed proteins. (**D**) KEGG analysis of *S. aureus* after treatment with KRP-203. (**E**) Protein-protein interaction network analysis of differentially expressed proteins between the KRP-203 treatment group and the DMSO group in *S. aureus* based on the KEGG database. Rectangles represent GO/KEGG pathways, with colors indicating the significance of the *P*-values. Circles represent proteins or genes, with colors representing fold changes.

Quantitative label-free proteomic analysis investigated the global proteomic response of *S. aureus* SA113 treated with KRP-203 during the exponential growth phase. In total, 1,259 proteins were confidently identified (matched peptides ≥1, and FDR < 0.01) and quantified. When compared with the control group, 438 proteins exhibited significantly different expression levels (≥|2|-fold change, *P* ≤ 0.05), including 232 upregulated and 206 downregulated proteins in the KRP-203 treatment group (Fig. 5A and B). Detailed information on the functional proteins with significantly different expression levels is listed in Table S2. Additionally, functional analysis of proteins with differentially expressed levels was conducted according to the gene ontology (GO) terms of biological process, molecular function, and cellular component (Fig. 5C). The results suggested that functional proteins with different expression levels in KRP-203-treated *S. aureus* could be classified as rRNA binding, structural molecule activity, and structural constituent of ribosome.

Moreover, KEGG analysis showed that metabolic pathways involved in the proteins with differential expression levels were enriched in glycolysis, microbial metabolism, cysteine and methionine metabolism, degradation of aromatic compounds, and pyruvate metabolism (Fig. 5D). Protein-protein interaction (PPI) network analysis was constructed using the STRING database (Fig. 5E). The results suggested that interactions among Type VII secretion system extracellular protein A esxA, thioredoxin trxA, anti-sigma-B factor antagonist rsbV, gamma-hemolysin component C, and lipase 2. These proteins were primarily involved in metabolic pathways associated with cell redox homeostasis, cytolysis, and lipid catabolic processes. Upregulated proteins were enriched in small ribosomal subunit protein uS14A rpsN, pyridoxal 5′-phosphate synthase subunit PdxT, and 1,4-dihydroxy-2-naphthoate octaprenyltransferase menA. These proteins primarily participated in metabolic pathways associated with translation, glutamine catabolic process, and menaquinone biosynthetic process. Critically, the pore-forming toxin component HlgC (4.82-fold downregulation) contributes to *S. aureus* pathogenicity by targeting host cell membranes. As part of the γ-hemolysin complex (HlgCB), HlgC binds phagocyte receptors (C5aR/C5L2) and inserts β-barrel pores into lipid bilayers, inducing osmotic lysis and Ca²^+^ dysregulation (18). Our results demonstrate that KRP-203 may exert its antibacterial activity against *S. aureus* by disrupting membrane integrity, a mechanism phenocopied by functional inhibition of HlgC-mediated pore formation. This membrane-targeting action underpins KRP-203’s bactericidal efficacy.

### Antibacterial mechanism of KRP-203 against *S. aureus*

**Fig 6:**
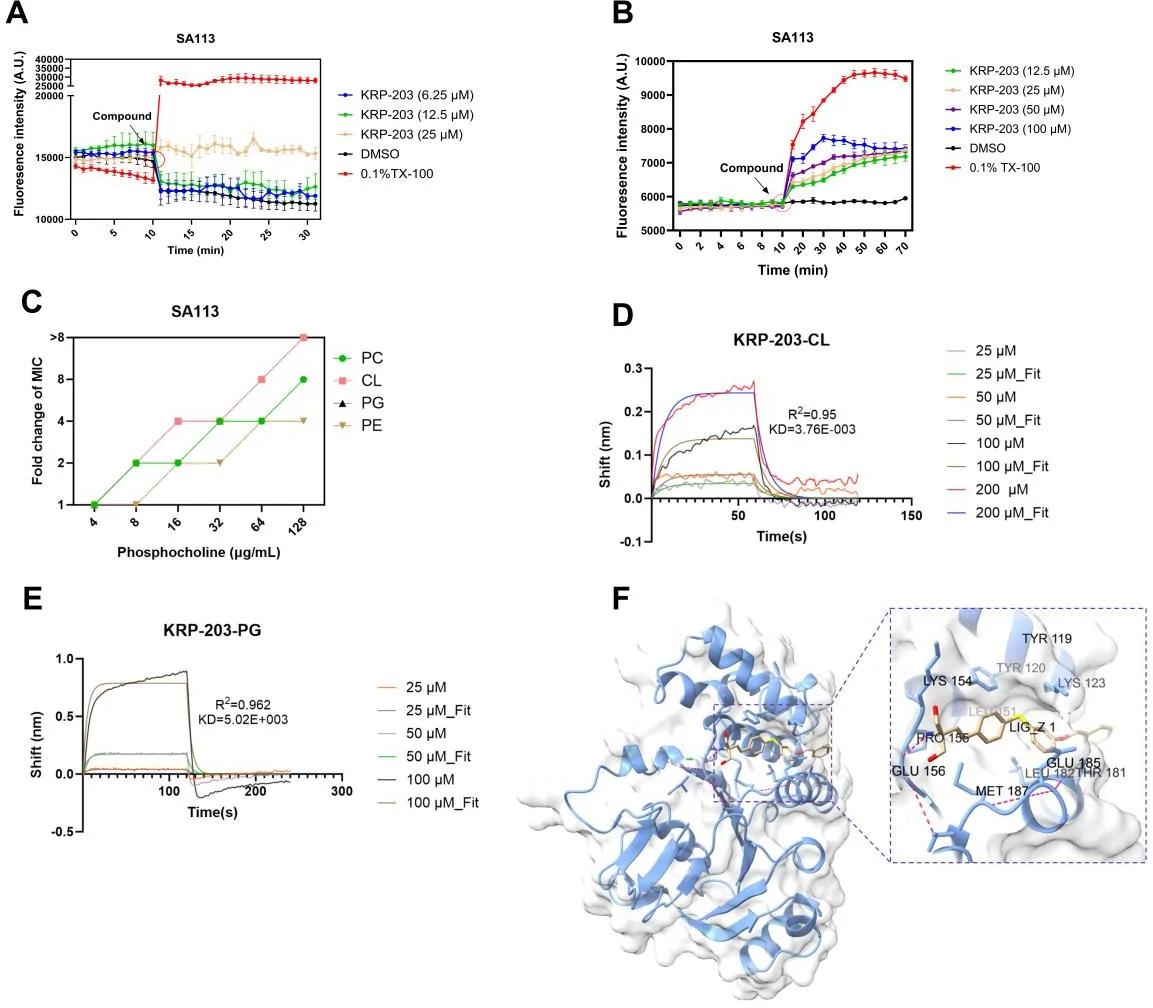
Effect of KRP-203 on the disruption of bacterial cytoplasmic membrane. (**A and B**) Membrane depolarization (**A**) and permeability (**B**) by KRP-203 against *S. aureus* SA113. (**C**) Membrane phospholipid neutralization experiment of *S. aureus* SA113. (**D and E**) Kinetic analysis by BLI of the binding of KRP-203 to cardiolipins (CL) and phosphatidylglycerol (PG). (**F**) Predicted molecular docking of KRP-203 in the glnQ-binding pocket.

To examine the influence of KRP-203 on the *S. aureus* cell membrane, the destructive effect of KRP-203 on cell membrane depolarization and membrane permeability was assessed using DiBAC4(3) and propidium iodide (PI) dye assays, respectively. The fluorescence intensity of *S. aureus* SA113 after DiBAC4(3) labeling was used to assess the depolarizing effect of substances on the cell membrane. KRP-203 showed a depolarizing impact on the cell membrane at doses of 1× MIC or 2× MIC (Fig. 6A), and KRP-203 treatment at 4× MIC raised PI fluorescence intensity in *S. aureus* SA113 by 1.23-fold compared with the control group (Fig. 6B). In general, the data clearly showed that KRP-203 rapidly disrupted *S. aureus* cell membrane permeability in a dose-dependent manner.

Membrane phospholipids are key components of *S. aureus* cell membranes. To further examine KRP-203’s antibacterial mechanism against *S. aureus*, four membrane phospholipids—PC, CL, PG, and PE—were exogenously introduced to bacterial culture conditions, and their effects on the MIC value of KRP-203 in *S. aureus* SA113 were detected. It was found that adding PC or CL at doses of ≥ 32 µg/mL enhanced the MIC value of KRP-203 against *S. aureus* SA113 by more than fourfold (Fig. 6C). Notably, the BLI kinetic analysis in this investigation also found strong binding interaction between KRP-203 and CL as well as KRP-203 and PG (Fig. 6D and E). Furthermore, the binding potential of glutamine transport ATP-binding protein glnQ with KRP-203 was predicted using molecular docking experiments (Fig. 6F). The results showed that KRP-203 may attach to glnQ in a favorable configuration, with a low binding energy of −8.7 kcal/mol. A lower score number (with a higher absolute value of the negative value) indicates a stronger binding force, whereas an affinity score of less than −7 kcal/mol indicates favorable binding. All together, these findings validated KRP-203’s ability to effectively attach to cell membrane or bind with proteins that is related with *S. aureus* cell membrane formation or metabolism. As a result, it was postulated that KRP-203’s antibacterial mechanism could be mediated via cell membrane-related pathways in *S. aureus*.

## DISCUSSION

Owing to their ubiquitous presence and extraordinary resistance in harsh circumstances, multidrug-resistant *Staphylococcus aureus* have emerged as one of the most common bacteria responsible for both hospital-acquired and community-associated illnesses (19). This expanding threat highlights the critical need to identify novel and effective antimicrobial drugs with diverse modes of action that can overcome existing resistance barriers (20). Drug repurposing, or reevaluating clinically approved compounds for novel antibacterial applications, is a viable and effective approach to speed up therapeutic development in this context while cutting down on the time and expense involved with conventional drug discovery pipelines (21). Mocravimod is an oral S1PR regulator that can prevent lymph organs from sending the necessary signal to stop affected cells from moving to hematopoietic tissue that is not lymphatic, and has potential use in cancer research (22). Clinically, mocravimod has been generally well tolerated, with reported host-related adverse effects primarily reflecting its mechanism of action, including dose-dependent lymphopenia and transient bradycardia, which are typically reversible and manageable (23). Importantly, clinical studies in the hematopoietic stem cell transplantation setting indicate that mocravimod does not impair donor cell engraftment or compromise graft-versus-leukemia effects, supporting an overall favorable safety profile despite potential drug-drug interactions involving CYP3A4/P-gp pathways (24). It has been found as a new antibacterial candidate by us with strong activity against *Staphylococcus aureus*, including methicillin-sensitive and methicillin-resistant clinical isolates. Given the pronounced differences in cell envelope architecture and resistance mechanisms between gram-positive and gram-negative bacteria, this study deliberately focused on *Staphylococcus aureus* as a clinically dominant gram-positive pathogen. The antibacterial activity of mocravimod against gram-negative organisms was therefore beyond the scope of the current work and will be explored in future studies. *In vitro*, KRP-203 demonstrated faster bactericidal kinetics than traditional antibiotics like vancomycin, eradicating planktonic *S. aureus* populations completely within 2 h at the concentration of 8× MIC. Importantly, this chemical inhibited biofilm formation at sub-inhibitory doses and effectively eradicated bacteria embedded in mature biofilms. These findings are clinically significant since biofilm-associated infections frequently underlie chronic and recurrent disease patterns and are resistant to traditional treatments (8, 25). However, KRP-203’s relatively high MIC values compared with first-line antibiotics may limit its systemic use unless local or topical formulations can be adjusted to achieve higher concentrations at the site of infection.

Despite KRP-203’s promising activity profile, the development of *S. aureus* resistance after continuous treatment indicates that bacterial adaptability remains a possible concern. After 60 generations of selective pressure, the *S. aureus* YUSA145 strain with an eightfold higher MIC was identified. The whole-genome sequencing of this KRP-203-induced resistant clone revealed three nonsynonymous mutations in genes involved in DNA recombination (rarA), amino acid transport (glnQ), and branched-chain amino acid metabolism (BCAT). Among them, glnQ is the hypothesized ATP binding subunit of an L-glutamine ABC transporter complex and it comprises characteristic patterns conserved in ATP-binding cassette (ABC) domains, linking ATP hydrolysis to glutamine translocation across the bacterial cytoplasmic membrane (26). Although there is limited evidence tying glnQ to membrane structural changes, its functional coordination with membrane-embedded permeases (such as glnP) suggests that glnQ mutations or disruptions may degrade membrane-associated transport dynamics and modify membrane potential (27). Furthermore, amino acid transport systems are becoming recognized for their indirect role in biofilm creation, as glutamine uptake influences nitrogen metabolism, intracellular signaling, and stress adaptation—all of which are important for biofilm maturation and persistence (28). Previous research has demonstrated that disrupting ABC transporters can lower biofilm biomass and alter extracellular matrix composition in numerous bacterial species (29), hinting that GlnQ may play a role in biofilm development or stability in *S. aureus*. Besides, BCAT is a PLP-dependent enzyme that reversibly transfers an amino group from branched-chain amino acids (valine, leucine, isoleucine) to α-ketoglutarate, resulting in their respective keto acids and glutamate (30). Bacteria convert keto acids into branched-chain acyl-CoAs, which act as precursors for branched-chain fatty acid synthesis (31). These fatty acids are absorbed into membrane lipids, which influence membrane fluidity and structure. All in all, both glnQ and BCAT regulate intracellular nitrogen metabolism and membrane lipid production. These findings indicate that bacterial resistance to KRP-203 may include changes in membrane homeostasis or adaptive shifts in amino acid usage, which could compensate for KRP-203-induced cellular damage. Although these genes are not classical antibiotic resistance determinants, their involvement is biologically plausible given their roles in membrane-associated transport and lipid metabolism. However, no direct validation of these gene activities in resistance development, such as complementation or knock-out experiments, was undertaken, and this should be addressed in future research.

Quantitative proteomic analysis provides a systems-level perspective on *S. aureus* response to mocravimod treatment, enabling a deeper understanding of how this compound perturbs key cellular processes and supports its potential antibacterial mechanism. In this study, the LC/MS-based proteomics data demonstrated the impact of KRP-203 on the global expression of the proteins in *S. aureus*, including 206 significantly downregulated proteins with enriched functional categories implicating translational machinery, redox regulation, and metabolic processes. It is worth noting that the pore-forming cytotoxin HlgC, a major virulence factor in *S. aureus*, was significantly downregulated after KRP-203 treatment. HlgC, the S-component of the γ-hemolysin CB complex (HlgCB), binds exclusively to the phagocyte complement receptors C5aR1 and C5L2. Once docked, it assembles with HlgB into octameric β-barrel pores within the lipid bilayer, resulting in fast Ca^2+^ influx, osmotic imbalance, and cell lysis (32). We hypothesized that KRP-203 compromises *S. aureus* membranes in a manner similar to genetic or functional inhibition of HlgC pore formation, suggesting that blocking HlgC’s activity could be a viable antibacterial strategy. Furthermore, GO and KEGG analyses revealed that upregulated proteins were enriched in pathways, such as menaquinone production and glutamine metabolism, which could indicate stress responses or compensatory adaptations to maintain energy balance and redox equilibrium. While our findings suggest that KRP-203 stress causes global cellular reprogramming, more mechanistic research is needed to determine the precise regulatory mechanisms and their direct relation to medication action.

From the results of the previous proteomics and whole genome sequencing, it can be seen that KRP-203’s antibacterial mechanism appears to be membrane-targeting. Our membrane depolarization and permeability experiments revealed a dose-dependent breakdown of cytoplasmic membrane integrity. Furthermore, membrane phospholipid supplementation drastically reduced KRP-203 efficacy, and biolayer interferometry validated KRP-203’s specific binding to CL and PG, both of which are important components of the *S. aureus* inner membrane (33). This lipid-binding property is consistent with the suggested membrane disruption mechanism. Furthermore, molecular docking research suggested a high binding affinity between KRP-203 and glnQ, implying that KRP-203 may interfere with membrane-associated transport systems. These observations are logically consistent with a membrane-targeting antibacterial mechanism. However, it is unclear whether membrane binding is the first step in bacterial killing or a result of metabolic disturbance. Time-resolved assays and improved imaging techniques (for example, cryo-EM or single-particle tracking) will be required to determine the temporal sequence and specificity of KRP-203 activity at the molecular level.

Several limitations to this study should be addressed. First, while KRP-203’s *in vitro* bactericidal and anti-biofilm characteristics were clearly proven, its *in vivo* efficacy, pharmacokinetics, and tissue distribution in infection models remain unknown. Given its high MICs compared with several traditional antibiotics, achieving adequate drug concentration at infection sites may be a limiting problem for systemic uses. In our early screening, we discovered a structural analog of KRP-203 with remarkably low MIC values against a wide range of gram-positive bacteria. This finding suggests that structural modification of KRP-203, or its combination with other antimicrobial agents, could increase its efficacy and lower the MIC necessary for effective suppression of *Staphylococcus aureus*. Future research is needed to investigate these methodologies and improve the antibacterial activity of KRP-203-based compounds. Second, while resistance-associated mutations and proteome alterations were discovered, direct functional causality between these molecular changes and resistance development was not experimentally validated. Gene editing, lipidomic profiling, and real-time metabolomics are all complementary approaches that could improve mechanistic understanding. Third, while KRP-203 exhibited pronounced inhibitory effects on biofilm formation and activity at the phenotypic level, the precise molecular mechanisms underlying this antibiofilm activity remain unclear. Although our data suggest that membrane perturbation plays a central role, it is currently unknown which specific stages of biofilm development like initial adhesion, maturation, or maintenance are primarily affected. Future studies employing transcriptional analyses of adhesion- and biofilm-associated genes, protein-level validation, and time-resolved biofilm assays will be critical to delineate the mechanistic basis of KRP-203-mediated biofilm inhibition. Finally, although molecular docking and biolayer interferometry supported the interaction of KRP-203 with membrane phospholipids and membrane-associated proteins, direct biophysical evidence of membrane insertion or destabilization was not obtained. Advanced biophysical techniques, such as surface plasmon resonance, liposome leakage assays, or high-resolution imaging approaches, should be applied in future work to further validate the proposed membrane-targeting mechanism.

This study makes several important contributions to the understanding and development of repurposed antibacterial agents against *Staphylococcus aureus*. First, we provide the first systematic evidence that KRP-203, a clinically approved S1PR modulator, exhibits robust antibacterial and antibiofilm activity against both MSSA and MRSA clinical isolates. This expands the antibacterial potential of immunomodulatory agents and highlights drug repurposing as a viable strategy to accelerate antimicrobial discovery. Second, by integrating phenotypic assays with whole-genome sequencing, quantitative proteomics, membrane functional assays, and lipid-binding analyses, this work offers a multidimensional mechanistic framework suggesting that KRP-203 primarily exerts its antibacterial effects through disruption of bacterial membrane homeostasis and interference with membrane-associated metabolic and virulence pathways. The identification of resistance-associated mutations and stress-responsive proteomic signatures further provides insight into bacterial adaptive responses under sustained drug pressure. Third, the demonstrated antibiofilm activity of KRP-203 addresses a critical unmet clinical need, as biofilm-associated *S. aureus* infections are notoriously difficult to eradicate using conventional antibiotics. Although the precise molecular basis of biofilm inhibition requires further investigation, our findings establish membrane perturbation as a plausible upstream mechanism linking antibacterial and antibiofilm effects.

In conclusion, our investigation provides the first evidence that KRP-203 has antibacterial and anti-biofilm activity against *S. aureus*, possibly via membrane-targeting mechanisms and interference with metabolic and virulence pathways. The detection of resistance-associated mutations, as well as the proteomics results, provide information about the drug’s mode of action and bacterial adaptation. These findings promote future research into KRP-203 as a repurposed antibacterial candidate, particularly for MRSA- and MSSA-associated infections. Continued efforts to clarify its mechanism, increase its potency, and demonstrate its *in vivo* efficacy are required to facilitate its clinical implementation.

## MATERIALS AND METHODS

### Bacterial isolates and growth conditions

Clinical isolates used in this study included methicillin-resistant *Staphylococcus aureus* (MRSA, *n* = 20), methicillin-sensitive *S. aureus* (MSSA, *n* = 19), *Staphylococcus epidermidis* (*n* = 22), *Enterococcus faecium* (*n* = 19), and *Enterococcus faecalis* (*n* = 21). These strains were obtained from individual patients at Shenzhen Nanshan People’s Hospital between 2011 and 2015 and were isolated from various clinical specimens including urine, blood, pus, sputum, and secretions, as previously described (34, 35). Species identification was performed using MALDI-TOF mass spectrometry (IVD MALDI Biotyper, Bruker, Karlsruhe, Germany). *E. faecalis* ATCC 29212, OG1RF, and *S. aureus* USA300 were used as quality control strains. For routine culture, *S. aureus* and *E. faecalis* strains were grown in tryptic soy broth (TSB) with or without 0.5% glucose supplementation (TSBG) at 37°C with shaking at 220 rpm. All bacterial strains were preserved in TSB containing 15% glycerol at −80°C for subsequent use.

### Minimum inhibitory concentration (MIC) determination

MIC values were determined by the broth microdilution method using cation-adjusted Mueller–Hinton broth (CAMHB), following the Clinical and Laboratory Standards Institute (CLSI) guidelines (M100, 29th edition). Bacterial suspensions were diluted 1:1,000 in CAMHB and dispensed into 96-well microtiter plates (Nest, China), followed by exposure to serial twofold dilutions of KRP-203 (ranging from 1.56 to 200 μM). Each well contained 200 μL of total volume. Controls consisted of CAMHB containing equivalent concentrations of KRP-203 dissolved in DMSO. After 24 h of incubation at 37°C, MICs were defined as the lowest drug concentration that completely inhibited visible bacterial growth. All compounds, including a library of FDA-approved clinical medicines, vancomycin (VAN), and linezolid (LZD), were obtained from MedChemExpress (Shanghai, China).

### Bacterial growth curve assay

Bacterial growth kinetics were assessed, as previously described (36), with modifications. Overnight cultures of *S. aureus* strains SA113 and CHS101 were diluted 1:1,000 into fresh TSB medium. KRP-203 was added at concentrations ranging from 1× to 1/16× MIC via twofold serial dilutions. Equal volumes (200 μL each) of bacterial suspension and drug solution were mixed in triplicate in the wells of a Bioscreen C system (Turku, Finland). Wells without KRP-203 served as growth controls. The optical density at 600 nm (OD_600_) was recorded at 1-h intervals for 16 h. Each assay was independently repeated three times.

### Time-Kill kinetic assay

The bactericidal activity of KRP-203 was assessed using a time-kill kinetics approach, as described previously (37), with slight modifications. Overnight cultures of *S. aureus* strains SA113 and YUSA145 were diluted 1:200 into fresh TSB medium and incubated at 37°C with shaking (220 rpm) until they reached logarithmic phase. Cell density was adjusted using sterile 0.9% NaCl to match a 0.5 McFarland standard (~1.0 × 10⁸ CFU/mL), then diluted 1:100 into TSB. KRP-203 was added at concentrations of 1×, 2×, 4×, and 8× MIC, and vancomycin was tested at 4× MIC. Cultures were incubated with shaking at 220 rpm and 37°C. At 2, 4, 6, and 24 h, 1-mL aliquots were withdrawn and centrifuged at 13,500 × *g* for 5 min. The supernatant was discarded, and the bacterial pellets were washed twice with 1 mL sterile 0.9% NaCl and resuspended. Following serial dilutions, 100 μL of each suspension was plated on TSA and incubated at 37°C for 24 h to determine viable cell counts. All experiments were conducted in triplicate.

### Biofilm formation assay

The inhibitory effect of KRP-203 on *S. aureus* biofilm formation was assessed using a semi-quantitative microtiter plate method, as described previously (38). *S. aureus* strains CHS655, CHS684, CHS736, CHS791, SA113, YUSA111, YUSA134, YUSA135, YUSA139, YUSA145, and CHS101 were cultured overnight in TSB, diluted 1:200 into fresh TSB, and grown to logarithmic phase. The cultures were then adjusted to a 0.5 McFarland standard (~1.0 × 10⁸ CFU/mL) using sterile 0.9% NaCl, followed by a 1:100 dilution into TSB supplemented with 0.25% glucose (TSBG). Aliquots of bacterial suspension (100 μL/well) were added to sterile 96-well microtiter plates along with equal volumes of KRP-203 diluted to final concentrations of 1/2×, 1/4×, and 1/8 × MIC. Plates were incubated at 37°C for 24 h. Untreated wells served as positive controls. After incubation, wells were washed three times with deionized water to remove planktonic cells. Biofilms were fixed with 95% methanol, stained with 1% crystal violet (100 μL/well, 20 min), and rinsed. Absorbance at 570 nm was measured using a microplate reader (BioTek, USA). All experiments were independently repeated three times.

### Quantification of adherent bacteria in biofilms

The effect of KRP-203 on bacterial adherence within biofilms was evaluated using a colony count method. A 1:500 dilution of bacterial culture in TSBG was added to 24-well plates (1 mL per well). KRP-203 was added at final concentrations of 1×, 1/2×, 1/4×, and 1/8× MIC, and the plates were incubated at 37°C for 24 h. Following incubation, the supernatant was carefully aspirated, and wells were washed twice with sterile saline to remove non-adherent cells. Residual adherent bacteria were resuspended and plated on TSB agar for viable colony enumeration. Each experiment was performed in triplicate.

### Membrane depolarization and permeability assay

Membrane integrity and potential disruption by KRP-203 were evaluated using a fluorescence-based assay, as previously reported (37). Overnight cultures of *S. aureus* SA113 were harvested and adjusted to ~10⁷ CFU/mL in HEPES buffer (5 mM HEPES, 20 mM glucose, pH 7.4). The bacterial suspensions were incubated with 1 μM propidium iodide (PI; excitation 217 nm, emission 340 nm) or 1 μM DiBAC4(3) for 2 h at 37°C. Potassium chloride was added to a final concentration of 0.1 M to equilibrate intra- and extracellular K^+^ concentrations. After fluorescence stabilization, KRP-203 was introduced to final concentrations of 1×, 2×, and 4× MIC. Fluorescence intensity was continuously monitored using a Cytation 1 cell imaging multimode reader (BioTek, USA). Wells containing 0.1% Triton X-100 and no drug treatment were used as positive and negative controls, respectively.

### Bacterial whole-genome sequencing

To induce resistance, *S. aureus* strain YUSA145 was cultured under escalating concentrations of KRP-203, starting at 3.125 μM and increasing every 5 days for a total of 60 days, until a final concentration of 8× MIC was reached. On day 60, some KRP-203-tolerant clones were isolated and passaged on TSA plates without drug pressure for five successive generations. A clone that consistently survived at 8× MIC was designated as the KRP-203-tolerant derivative for subsequent genetic analysis. Genomic DNA from this derivative was extracted as previously described and subjected to whole-genome sequencing (39). Library preparation was performed using the Nextera kit, and sequencing was conducted using the Illumina HiSeq platform (Novogene Co., Ltd., Beijing, China). Raw reads were aligned to the YUSA145 reference genome using BWA-MEM (v0.7.5a) with default parameters. Variants including single nucleotide polymorphisms (SNPs) and indels were identified using MUMmer (v3.23). The sequencing data for the KRP-203-induced *S. aureus* YUSA145 derivative have been deposited in the NCBI BioProject database under accession number PRJNA1121100 (NCBI: https://www.ncbi.nlm.nih.gov/bioproject/1121100).

### Sample preparation for proteomics

Quantitative proteomic sample preparation was carried out based on a previously described protocol with modifications (40). *Staphylococcus aureus* SA113 was grown overnight in TSB, then diluted 1:200 in fresh medium and cultured to mid-exponential phase (OD_600_ ≈ 0.5–0.8). KRP-203 was added to the cultures at a final concentration of 1/2× MIC. Cultures treated with an equivalent volume of DMSO served as controls. Each condition was tested in triplicate. Following drug treatment, the cultures were incubated at 37°C for 2 h with shaking at 200 rpm. Cells were collected by centrifugation at 5,000 × *g* for 10 min at 4°C and washed three times with pre-chilled PBS. Bacterial pellets were then lysed in RIPA buffer (1% Triton X-100, 1% sodium deoxycholate, 0.1% SDS) supplemented with a protease inhibitor cocktail (Roche, Cat. No. 05892970001, Basel, Switzerland). Cell disruption was performed by three cycles of mechanical homogenization using 0.1-mm diameter glass beads, followed by centrifugation at 12,000 × *g* for 20 min at 4°C. The resulting supernatants were collected for protein quantification and subsequent proteomic analysis. Protein concentrations were determined using the Pierce Micro BCA Protein Assay Kit (Thermo Fisher Scientific, Cat. No. 23,227, MA, USA). For digestion, 100 μg of protein per sample was reduced with 10 mM dithiothreitol (DTT; Sigma-Aldrich, St. Louis, MO) at 70°C for 1 h and alkylated with 50 mM iodoacetamide (IAA; Sigma-Aldrich) for 15 min at room temperature in the dark. Samples were buffer-exchanged with 0.5 M ammonium bicarbonate using 10 kDa Amicon Ultra centrifugal filters (Millipore, Billerica, MA), and proteins were digested overnight with trypsin (Promega, Madison, WI) at a 1:50 enzyme-to-substrate ratio at 37°C. Peptides were lyophilized and stored at −80°C until further analysis.

### Nano LC-MS/MS analysis for proteomics

Dried peptides were reconstituted in 30 μL of 0.1% formic acid, and 4 μL of each sample was injected into an UltiMate 3000 RSLC nano liquid chromatography system coupled with a Q Exactive Plus mass spectrometer via a nano-electrospray ionization (NSI) interface. Samples were first trapped on a C18 pre-column (100 μm × 20  mm, Acclaim PepMap 100, 3 μm), then separated on an analytical C18 column (75 μm × 250  mm, Acclaim PepMap RSLC, 2 μm). The mobile phase consisted of solvent A (0.1% formic acid in water) and solvent B (80% acetonitrile in 0.1% formic acid). The elution program began with 5% B for 5 min, followed by a linear gradient from 5% to 38% B over 50 min, a ramp to 95% B over 2 min, and a hold at 95% B for 8 min. The flow rate was maintained at 300 nL/min. Full MS scans (MS1) were acquired over a mass range of m/z 350–1,500 at a resolution of 70,000. The top N precursor ions (excluding singly charged species and ions with undefined charge states) were selected for MS/MS analysis (MS2) at a resolution of 17,500, with a maximum injection time of 50 ms. Dynamic exclusion was set to 25 seconds to avoid repeated scanning of the same precursor ion.

### Bioinformatics analysis for proteomics

Mass spectrometry data were processed using Proteome Discoverer software version 2.4 (Thermo Fisher Scientific), employing the Sequest HT search engine against the *S. aureus* strain NCTC 8325/PS 47 Uniprot reference proteome (UP000008816.fasta, 2,889 entries; downloaded on 20 February 2021). Protein identifications were filtered based on a ≥2-fold change and a *P*-value < 0.05 in at least two technical replicates to define significantly up- or downregulated proteins. Differentially expressed proteins were functionally annotated using the OMICSBEAN online platform (http://www.omicsbean.com). Analyses included Gene Ontology (GO) classifications for biological process, molecular function, and cellular component, as well as pathway mapping via the Kyoto Encyclopedia of Genes and Genomes (KEGG). Protein–protein interaction (PPI) networks were also generated using the OMICSBEAN tool.

### Antibacterial activity of KRP-203 in the presence of phospholipids

To investigate how phospholipids influence the antibacterial activity of KRP-203, a checkerboard assay was performed, as described previously (41). Phospholipids including phosphatidylcholines (PC), cardiolipins (CL), phosphatidylglycerol (PG), and phosphatidylethanolamine (PE) were dissolved in methanol and diluted to final concentrations ranging from 4 to 128 μg/mL. In 96-well plates, phospholipids were titrated along the vertical axis and different concentrations of KRP-203 along the horizontal axis. *Staphylococcus aureus* SA113 was inoculated into each well at 1 × 10⁶ CFU per well in TSB medium. After 24 h of incubation at 37°C, bacterial growth was assessed by measuring OD_600_ using a microplate reader. The MIC of KRP-203 in combination with phospholipids was evaluated based on changes in optical density relative to controls. All phospholipids were obtained from Aladdin (Shanghai, China).

### Biolayer interferometry (BLI) assay

Binding affinity between KRP-203 and the phospholipids CL and PG was assessed by biolayer interferometry using a Gatorprime system (Gator Bio, San Francisco, USA), as previously described (34). Biotin-labeled phospholipids were immobilized onto streptavidin-coated biosensors, which were pre-equilibrated in kinetic buffer (PBS containing 0.05% BSA and 0.01% Tween-20). KRP-203 was prepared at various concentrations and applied to the biosensor tips. Measurements were conducted at 30°C in 96-well black microplates, with 300 μL total volume per well. To control for background signal and nonspecific interactions, parallel biosensors were incubated in buffer without protein. Data acquisition and kinetic analysis were performed using Gatorprime software, applying double reference subtraction. Association and dissociation rates (K_on_ and K_off_) were calculated to determine the equilibrium dissociation constant (Kd).

### Molecular docking simulation

Molecular docking was performed to predict the interaction between KRP-203 and the glutamine transport ATP-binding protein glnQ (Uniprot ID: O34677). The three-dimensional structure of glnQ was obtained from AlphaFoldDB (42) and prepared using Schrödinger’s Protein Preparation Wizard, which included hydrogen atom addition, residue completion, and structural optimization of water molecules. The 3D structure of KRP-203 (CAS No. HY-13660) was retrieved from the PubChem database. Docking simulations were carried out according to established protocols (43). Binding pocket identification was achieved through structure-based cavity detection, and docking was performed using AutoDock Vina and Discovery Studio. The most favorable binding pose was selected based on the Vina binding affinity score (kcal/mol).

### Quantification and statistical analysis

All statistical analyses were conducted using GraphPad Prism version 10.0. Data are reported as mean ± standard deviation (SD). Comparisons between groups were made using Student’s *t*-test. A *P*-value less than 0.05 was considered statistically significant (*P* < 0.05, *P* < 0.01, *P < 0.001). Unless otherwise noted, all experiments were performed in at least three biological replicates, yielding reproducible reslts.

## Supplementary Material

Reviewer comments

## SUPPLEMENTAL MATERIAL

Table S1 spectrum.02263-25-s0001.docx Specific MICs values of KRP-203 against various bacterial species.

Table S2 spectrum.02263-25-s0002.xlsx Protein quantification of significantly reduced or increased expression of SA113 after KRP-203 treatment.

OPEN PEER REVIEW reviewer-comments.pdf An accounting of the reviewer comments and feedback.

ASM does not own the copyrights to Supplemental Material that may be linked to, or accessed through, an article. The authors have granted ASM a non-exclusive, world-wide license to publish the Supplemental Material files. Please contact the corresponding author directly for reuse.
