# Supplementary material for: Mocravimod as a repurposing drug against clinical isolates of Staphylococcus aureus by targeting cell membrane
Source: Microbiol Spectr. 2026 Jul 27;14(9):e02263-25. doi: 10.1128/spectrum.02263-25 (PMC13532308; doi:10.1128/spectrum.02263-25)
Supplement: Supplementary file 1 [file reviewer-comments.pdf]

AMERICAN SOCIETY FOR MICROBIOLOGY | Microbiology Spectrum

Mocravimod as a repurposing drug against clinical isolates of *Staphylococcus aureus* by targeting cell membrane

Yuanyuan Tang, Yuqing Xia, Xuancheng Huang, Zhijian Yu, Duoyun Li, Shiqing Han, Peiyu Li, and Tieying Hou

Corresponding Author(s): Peiyu Li, Shenzhen Nanshan People's Hospital

Review Timeline:

Submission Date:	July 24, 2025
Editorial Decision:	November 4, 2025
Revision Received:	December 30, 2025
Accepted:	April 25, 2026

Editor: Kai Zhou

Reviewer(s): Disclosure of reviewer identity is with reference to reviewer comments included in decision letter(s). The following individuals involved in review of your submission have agreed to reveal their identity: Madhavi Annamanedi (Reviewer #1); Amjed alsultan (Reviewer #2)

Transaction Report:

(Note: With the exception of the correction of typographical or spelling errors that could be a source of ambiguity, letters and reports are not edited. The original formatting of letters and referee reports may not be reflected in this compilation.)

DOI: https://doi.org/10.1128/spectrum.02263-25

Re: Spectrum02263-25 (**Addressing antibiotic resistance in *Staphylococcus aureus* by repurposing Mocravimod with novel antibacterial and antibiofilm potential**)

Dear Dr. Peiyu Li:

Thank you for the privilege of reviewing your work. Below you will find my comments, instructions from the Spectrum editorial office, and the reviewer comments.

Please return the manuscript within 60 days; if you cannot complete the modification within this time period, please contact me. If you do not wish to modify the manuscript and prefer to submit it to another journal, notify me immediately so that the manuscript may be formally withdrawn from consideration by Spectrum.

Revision Guidelines
To submit your modified manuscript, log into the submission site at https://spectrum.msubmit.net/cgi-bin/main.plex. Go to Author Tasks and click the appropriate manuscript title to begin. The information you entered when you first submitted the paper will be displayed; update this as necessary. Note the following requirements:

• Upload point-by-point responses to the issues raised by the reviewers in a file named "Response to Reviewers," NOT in your cover letter.
• Upload a compare copy of the manuscript (without figures) as a "Marked-Up Manuscript" file.
• Upload a clean .DOC/.DOCX version of the revised manuscript and remove the previous version.
• Each figure must be uploaded as a separate, editable, high-resolution file (TIFF or EPS preferred), and any multipanel figures must be assembled into one file.
• Any supplemental material intended for posting by ASM should be uploaded with their legends separate from the main manuscript. You can combine all supplemental material into one file (preferred) or split it into a maximum of 10 files with all associated legends included.

For complete guidelines on revision requirements, see our Submission and Review Process webpage. Submission of a paper that does not conform to guidelines may delay acceptance of your manuscript.

Data availability: ASM policy requires that data be available to the public upon online posting of the article, so please verify all links to sequence records, if present, and make sure that each number retrieves the full record of the data. If a new accession number is not linked or a link is broken, provide Spectrum production staff with the correct URL for the record. If the accession numbers for new data are not publicly accessible before the expected online posting of the article, publication may be delayed; please contact production staff (Spectrum@asmusa.org) immediately with the expected release date.

Publication Fees: For information on publication fees and which article types are subject to charges, visit our website. If your manuscript is accepted for publication and any fees apply, you will be contacted separately about payment during the production process; please follow the instructions in that e-mail. Arrangements for payment must be made before your article is published.

ASM Membership: Corresponding authors may join or renew ASM membership to obtain discounts on publication fees. Need to upgrade your membership level? Please contact Customer Service at Service@asmusa.org.

The ASM Journals program strives for constant improvement in our submission and publication process. Please tell us how we can improve your experience by taking this quick Author Survey.

Thank you for submitting your paper to Spectrum.

Sincerely,
Kai Zhou
Editor
Microbiology Spectrum

Reviewer #1 (Comments for the Author):

Figure 1A and B positive control is required

Biofilm testing should have biofilm positive control strain to compare

Reviewer #2 (Comments for the Author):

Addressing antibiotic resistance in Staphylococcus aureus by repurposing Mocravimod with novel antibacterial and antibiofilm potential
In this study, the antibacterial activity of Mocravimod, a sphingosine-1-phosphate (S1P) receptor modulator, was evaluated against clinical isolates of Staphylococcus. The results demonstrated that Mocravimod exhibited significant antibacterial activity against the tested Staphylococcus strains. The observed antibacterial effect is presumed to arise from the compound's disruptive impact on bacterial cell membrane integrity. The study is primarily interested. However, the study was limited by the lack of assessment of Mocravimod's selectivity and cytotoxicity toward live eukaryotic cells at its minimum inhibitory concentration (MIC).
Following requests are necessary to be addressed to improve quality of the study.
Title:
- Although the topic is within the scope of the study, the claim "addressing antibiotic resistance in Staphylococcus aureus" appears overstated based on the presented data. It is recommended that the title be adjusted to more accurately reflect the study's primary focus, namely the evaluation of the antibacterial activity of Mocravimod against clinical isolates of Staphylococcus aureus.
Abstract:
- Line 21-22: Could you please clarify the basis for selecting Mocravimod to evaluate its antibacterial activity against this bacterium? Was the selection based on previous studies, clinical observations, or another rationale?
- Line 24 : Were the tested clinical strains evaluated for their antibiotic resistance profiles? Specifically, did you identify any multidrug-resistant (MDR) or extensively drug-resistant (XDR) isolates among them? This point needs clarification.
- Line 29: Please clarify how many samples were analyzed by whole-genome sequencing (WGS) and on what basis they were selected.
- Line 29: Additional details are needed regarding the quantitative proteomics analysis and how it was used to elucidate the antibacterial activity of Mocravimod against Staphylococcus. Please clarify the proteomic approach applied, the key protein expression changes observed, and how these findings support the proposed antibacterial mechanism.
- Line 32: How was the alteration in gene expression measured? Whole-genome sequencing (WGS) alone is not suitable for assessing expression profiles, so clarification of the methodology used for expression analysis is needed.
- In the study design, please clarify why the antibacterial activity of Mocravimod was evaluated only against Staphylococcus. What was the rationale for not including E. coli or other Gram-negative microorganisms for comparison?
- Line 37: A clear presentation of the study's key findings, along with its limitations and the need for further research, should be provided in this section.
- This section should be revised to provide a clear and logical narrative, beginning with the study's objectives, followed by a detailed, stepwise description of the methodology including the number of isolates and the specific methods employed and concluding with a coherent presentation of the results obtained.
Introduction:
- In this section, the chemical structure, biological activity, and associated pathways of Mocravimod should be thoroughly detailed, and any potential adverse effects on the host should be clearly discussed.

Results
- Line 117, This information should be included in the Abstract section.
- Line 117: The type of screening approach should be described in detail-specifically, whether it involved in silico analysis, computer-based screening, or a structural bioinformatics-based screening method.
- In this screening, no results were presented for Gram-negative bacteria, particularly E. coli. It should be clarified whether the study intentionally focused only on Staphylococcus and, if so, the rationale for this choice should be explained.
- Line 136 , Since the isolates exhibit varying minimum inhibitory concentrations (MICs) for different antibiotics, it is necessary to compare the effect of the tested agent alongside more than one antibiotic, rather than limiting the comparison to vancomycin alone.
- Line 145,Table 1: Did you compare the MIC of KRP-203 against Gram-positive bacterial isolates with its clinically applicable dosage?
- Line 164 : It is necessary to demonstrate the proposed mechanism by which KRP-203 inhibits biofilm formation or activity in the tested bacterial strains.

Discussion

- In need to explain if the result gotten is logical or some of them is unexpected
- Clearly explain the limitations of the study and recommend further research
- Include a section that highlights and explains the clear contributions and implications of this study.
-
Methods:
- Line 431: Please provide more details on the source of the bile samples, including the host species from which they were collected, as well as the methods used for bacterial isolation.

Addressing antibiotic resistance in Staphylococcus aureus by repurposing Mocravimod with novel antibacterial and antibiofilm potential

In this study, the antibacterial activity of Mocravimod, a sphingosine-1-phosphate (S1P) receptor modulator, was evaluated against clinical isolates of Staphylococcus. The results demonstrated that Mocravimod exhibited significant antibacterial activity against the tested Staphylococcus strains. The observed antibacterial effect is presumed to arise from the compound's disruptive impact on bacterial cell membrane integrity. The study is primarily interested. However, the study was limited by the lack of assessment of Mocravimod's selectivity and cytotoxicity toward live eukaryotic cells at its minimum inhibitory concentration (MIC).

Following requests are necessary to be addressed to improve quality of the study.

Title:

- Although the topic is within the scope of the study, the claim "addressing antibiotic resistance in Staphylococcus aureus" appears overstated based on the presented data. It is recommended that the title be adjusted to more accurately reflect the study's primary focus, namely the evaluation of the antibacterial activity of Mocravimod against clinical isolates of Staphylococcus aureus.

Abstract:

- Line 21-22: Could you please clarify the basis for selecting Mocravimod to evaluate its antibacterial activity against this bacterium? Was the selection based on previous studies, clinical observations, or another rationale?
- Line 24 : Were the tested clinical strains evaluated for their antibiotic resistance profiles? Specifically, did you identify any multidrug-resistant (MDR) or extensively drug-resistant (XDR) isolates among them? This point needs clarification.
- Line 29: Please clarify how many samples were analyzed by whole-genome sequencing (WGS) and on what basis they were selected.
- Line 29: Additional details are needed regarding the quantitative proteomics analysis and how it was used to elucidate the antibacterial activity of Mocravimod against Staphylococcus. Please clarify the proteomic approach applied, the key protein expression changes observed, and how these findings support the proposed antibacterial mechanism.
- Line 32: How was the alteration in gene expression measured? Whole-genome sequencing (WGS) alone is not suitable for assessing expression profiles, so clarification of the methodology used for expression analysis is needed.
- In the study design, please clarify why the antibacterial activity of Mocravimod was evaluated only against Staphylococcus. What was the rationale for not including E. coli or other Gram-negative microorganisms for comparison?
- Line 37: A clear presentation of the study's key findings, along with its limitations and the need for further research, should be provided in this section.
- This section should be revised to provide a clear and logical narrative, beginning with the study's objectives, followed by a detailed, stepwise description of the methodology including the number of isolates and the specific methods employed and concluding with a coherent presentation of the results obtained.

Introduction:

- In this section, the chemical structure, biological activity, and associated pathways of Mocravimod should be thoroughly detailed, and any potential adverse effects on the host should be clearly discussed.

Results

- Line 117, This information should be included in the Abstract section.
- Line 117: The type of screening approach should be described in detail—specifically, whether it involved in silico analysis, computer-based screening, or a structural bioinformatics–based screening method.
- In this screening, no results were presented for Gram-negative bacteria, particularly E. coli. It should be clarified whether the study intentionally focused only on Staphylococcus and, if so, the rationale for this choice should be explained.
- Line 136 , Since the isolates exhibit varying minimum inhibitory concentrations (MICs) for different antibiotics, it is necessary to compare the effect of the tested agent alongside more than one antibiotic, rather than limiting the comparison to vancomycin alone.
- Line 145,Table 1: Did you compare the MIC of KRP-203 against Gram-positive bacterial isolates with its clinically applicable dosage?
- Line 164 : It is necessary to demonstrate the proposed mechanism by which KRP-203 inhibits biofilm formation or activity in the tested bacterial strains.

Discussion

- In need to explain if the result gotten is logical or some of them is unexpected
- Clearly explain the limitations of the study and recommend further research
- Include a section that highlights and explains the clear contributions and implications of this study.
-

Methods:

- Line 431: Please provide more details on the source of the bile samples, including the host species from which they were collected, as well as the methods used for bacterial isolation.

Dear Editor and Reviewers,

We would like to thank you and the reviewers for your valuable time and insightful comments on our manuscript. We have carefully revised the manuscript according to the suggestions provided, and we believe that these revisions have improved the quality and clarity of our work.

Below, we provide a point-by-point response to each of the comments raised by the reviewers.

Reviewer 1's Comments:

Comment R1-1: Figure 1A and B positive control is required.
Response R1-1: We thank the reviewer for this helpful comment. We would like to clarify the experimental design and the definition of controls used in Figure 1A and B (now Figure 2A and B).

The purpose of this experiment was to evaluate the effect of KRP-203 on the growth of *Staphylococcus aureus* and to assess whether growth inhibition occurs in a concentration-dependent manner. In this context, the negative control corresponds to bacterial cultures grown in the absence of KRP-203. The positive control in this assay is the treatment with KRP-203 at 1 × MIC, which represents the minimal concentration required to completely inhibit bacterial growth. In Figure 2A and B, this condition is shown as the purple solid line and demonstrates full suppression of bacterial growth over the incubation period.

If the reviewer refers to a positive control using a clinically established antibiotic (e.g., vancomycin) for direct comparison, we respectfully believe that such an additional control is not essential for this specific experiment. The primary objective of the growth curve assay was not to compare the antibacterial efficacy of KRP-203 with that of standard-of-care antibiotics, but rather to confirm that KRP-203 itself effectively inhibits *S. aureus* growth and to visualize its dose-dependent inhibitory effect. Notably, treatment with KRP-203 at 1 × MIC already resulted in complete growth inhibition, which sufficiently demonstrates its antibacterial activity in this assay.

Moreover, the experimental design and presentation of growth curves without inclusion of an additional antibiotic control are consistent with previously published studies using similar methodologies (1-3). Therefore, we consider the current presentation of Figure 2A and B to be appropriate for the stated experimental purpose and do not believe that additional modifications are required.

Nevertheless, we appreciate the reviewer's suggestion and would be happy to further revise the figure or clarify the experimental design if our interpretation of this

comment does not fully address the reviewer's concern.

Comment R1-2: Biofilm testing should have biofilm positive control strain to compare.

Response R1-2: We thank the reviewer for this valuable suggestion. We would like to clarify how biofilm-positive strains were defined and selected in our study.

According to widely accepted criteria in the literature, biofilm formation is evaluated based on the optical density at 570 nm (OD_{570}) measured after crystal violet staining (4). Using this method, the cutoff value (Dc) is defined as twice the OD570 value of the negative control, and biofilm-forming capacity is classified as follows: strong biofilm producers (D>2Dc), weak biofilm producers (Dc≤D≤2Dc), and non-biofilm producers (D≤Dc). All assays were performed in triplicate, and mean values were used for classification.

In the present study, all *S. aureus* clinical isolates used in Figure 2A and B (now Figure 3A and B) including both MSSA and MRSA strains were pre-characterized for their biofilm-forming ability using this standard crystal violet assay. Based on OD_{570} measurements, all selected strains met the criteria for biofilm-positive strains. During the experiments, robust biofilm formation was consistently observed, as evidenced by visible adherent, whitish biofilm material at the bottom of the 96-well plates, with OD_{570} values typically exceeding 2.0.

Importantly, these strains have also been used as biofilm-positive isolates in our previously published studies, in which the same experimental procedures and evaluation criteria were applied. While absolute OD_{570} values obtained from untreated controls may vary slightly across different experiments or publications, potentially due to differences in incubation time, washing steps, or minor operational variations, this variability does not affect the classification of these strains as biofilm-positive.

Given that all tested strains in the current study are confirmed biofilm-forming isolates, and that the experimental aim was to assess the inhibitory effect of KRP-203 on biofilm formation rather than to compare biofilm formation across different reference strains, we believe that inclusion of an additional biofilm-positive reference strain is not strictly necessary for interpretation of the results presented in Figure 3.

Nevertheless, we appreciate the reviewer's comment and would be happy to further clarify this point in the manuscript or revise the experimental description if additional clarification is deemed helpful.

References

1. Wen Z, Zhao Y, Gong Z, Tang Y, Xiong Y, Chen J, Chen C, Zhang Y, Liu S, Zheng J. 2022. The mechanism of action of Ginkgolic acid (15: 1) against gram-positive bacteria involves cross talk with iron homeostasis. Microbiology spectrum 10:e00991-21.
2. Xiong Y, Wang R, Zheng J, Fang D, He P, Liu S, Lin Z, Chen X, Chen C, Shang Y. 2024. Discovery of novel dihydropyrrolidone-thiadiazole compound crosstalk between the YycG/F two-component regulatory pathway and cell membrane homeostasis to combat methicillin-resistant Staphylococcus aureus. European Journal of Medicinal Chemistry 277:116770.
3. He Y, Liu F, Li C, Wu J, Fan K, Wen Z, Li D, Yu Z, Hou T. 2025. The adaptive growth and mechanisms of Klebsiella pneumoniae under sucrose and glucose exposure. Microbiology Spectrum 13:e01603-25.
4. Hassan A, Usman J, Kaleem F, Omair M, Khalid A, Iqbal M. 2011. Evaluation of different detection methods of biofilm formation in the clinical isolates. Brazilian journal of infectious diseases 15:305-311.

Reviewer 2's Comments:

Comment R2-1: Although the topic is within the scope of the study, the claim "addressing antibiotic resistance in Staphylococcus aureus" appears overstated based on the presented data. It is recommended that the title be adjusted to more accurately reflect the study's primary focus, namely the evaluation of the antibacterial activity of Mocravimod against clinical isolates of Staphylococcus aureus.

Response R2-1: We sincerely thank the reviewer for this insightful and constructive comment. We agree that the original title may have overstated the extent.

In response to this suggestion, we have revised the title to more accurately reflect the primary scope and findings of our work. The title has been changed from "Addressing antibiotic resistance in *Staphylococcus aureus* by repurposing Mocravimod with novel antibacterial and antibiofilm potential" to "Mocravimod as a repurposing drug against clinical isolates of *Staphylococcus aureus* by targeting the cell membrane."

We believe that the revised title more precisely represents the core focus of the study, which is the evaluation of the antibacterial and antibiofilm activity of Mocravimod against clinical *S. aureus* isolates and the investigation of its membrane-targeting mechanism, without overextending claims regarding antibiotic resistance.

We appreciate the reviewer's valuable feedback, which has helped improve the clarity and accuracy of the manuscript.

Comment R2-2: Line 21-22: Could you please clarify the basis for selecting Mocravimod to evaluate its antibacterial activity against this bacterium? Was the selection based on previous studies, clinical observations, or another rationale?

Response R2-2: We thank the reviewer for this important question and the opportunity to clarify the rationale behind the selection of Mocravimod.

Mocravimod (KRP-203) was not selected based on prior reports of antibacterial activity or clinical observations related to bacterial infections. Instead, its antibacterial potential was identified through a screening of a focused library of FDA-approved clinical compounds purchased from MedChemExpress (MCE). This library was selected to facilitate drug repurposing efforts, leveraging compounds with well-characterized pharmacokinetic and safety profiles. During this preliminary screening, Mocravimod emerged as a compound exhibiting reproducible inhibitory activity against *Staphylococcus aureus*. Based on this initial observation, we subsequently performed systematic *in vitro* assays to evaluate its antibacterial and antibiofilm activities against both reference strains and clinical isolates, followed by mechanistic studies to explore its mode of action.

This screening-based selection process has been briefly described in the Introduction section of the manuscript, where we state: "**The antibacterial activity of KRP-203 was initially discovered by screening a specific library of FDA-approved clinical medicines.**" We appreciate the reviewer's comment, which has helped us improve the transparency and clarity of the study design.

Comment R2-3: Line 24 : Were the tested clinical strains evaluated for their antibiotic resistance profiles? Specifically, did you identify any multidrug-resistant (MDR) or extensively drug-resistant (XDR) isolates among them? This point needs clarification.
Response R2-3: We thank the reviewer for this important comment and appreciate the opportunity to clarify the antibiotic resistance profiles of the clinical isolates used in this study.

All clinical *Staphylococcus aureus* isolates included in this work were subjected to comprehensive antimicrobial susceptibility testing. Based on their resistance profiles, the isolates were classified as either methicillin-susceptible *S. aureus* (MSSA) or methicillin-resistant *S. aureus* (MRSA). Detailed strain classification is provided in the Supplementary Materials.

According to widely accepted definitions, multidrug-resistant (MDR) bacteria are defined as isolates resistant to at least three classes of antimicrobial agents, whereas extensively drug-resistant (XDR) bacteria are defined as isolates that remain susceptible to only one or two antimicrobial agents with potential activity (commonly including tigecycline and/or polymyxins). Based on these criteria, all MRSA isolates included in our study meet the definition of MDR strains.

Upon further examination of the antimicrobial susceptibility profiles, no extensively drug-resistant (XDR) isolates were identified among the tested MRSA strains. This information has now been explicitly indicated in the Supplementary Materials as "**MRSA (no XDR isolates)**" to improve clarity. We appreciate the reviewer's comment, which has helped us better clarify the resistance characteristics of the

clinical isolates used in this study.

Comment R2-4: Line 29: Please clarify how many samples were analyzed by whole-genome sequencing (WGS) and on what basis they were selected.

Response R2-4: We thank the reviewer for this important comment and the opportunity to clarify the WGS sample selection and experimental design.

Whole-genome sequencing was performed on a total of two *Staphylococcus aureus* strains: the parental strain YUSA145 and a KRP-203-induced tolerant derivative strain, designated YUSA145N60. The rationale for selecting these two strains is described in detail in the Results section entitled "**Genetic mutation in KRP-203-induced *S. aureus***," although it was not elaborated in the Abstract due to space limitations.

Briefly, the parental strain YUSA145 was continuously passaged *in vitro* under increasing selective pressure of KRP-203, starting from 1/2 × MIC and gradually increasing the drug concentration. After 60 sequential passages, a tolerant strain (YUSA145N60) was obtained, exhibiting an eight-fold increase in the MIC of KRP-203 (from 12.5 µM to 100 µM). This induced strain was selected for WGS to identify genetic alterations potentially associated with reduced susceptibility to KRP-203. Whole-genome sequencing data from YUSA145N60 were then compared with those of the parental YUSA145 strain, for which genome sequencing had been previously completed, using single-nucleotide polymorphism (SNP) analysis. The primary objective of this approach was to identify candidate mutations that may contribute to KRP-203 tolerance and to provide mechanistic insights into potential antibacterial targets or pathways affected by KRP-203, thereby guiding subsequent functional validation experiments. We appreciate the reviewer's comment, which has helped us clarify the rationale and scope of the WGS analysis in this study.

Comment R2-5: Line 29: Additional details are needed regarding the quantitative proteomics analysis and how it was used to elucidate the antibacterial activity of Mocravimod against Staphylococcus. Please clarify the proteomic approach applied, the key protein expression changes observed, and how these findings support the proposed antibacterial mechanism.

Response R2-5: We thank the reviewer for this detailed and constructive comment. We have clarified the proteomic workflow, key findings, and their mechanistic relevance as outlined below.

First, regarding the proteomic approach, the quantitative proteomics analysis consisted of three main steps: (i) sample preparation and protein digestion, (ii) nano-liquid chromatography coupled with tandem mass spectrometry (Nano LC-MS/MS), and (iii) bioinformatics analysis, including protein identification, quantification, and functional enrichment. Detailed experimental procedures, parameters, and data processing methods for each step are fully described in the

Materials and Methods section.

Second, concerning the key protein expression changes, label-free quantitative proteomics identified a broad set of differentially expressed proteins (both upregulated and downregulated) in *S. aureus* treated with Mocravimod. Proteins exhibiting the most significant expression changes are listed in **Supplementary Table S2**.

Third, regarding how these findings support the proposed antibacterial mechanism, the proteomic data provide molecular-level evidence that links Mocravimod treatment to membrane-associated antibacterial effects in *S. aureus*:

1. **Target-associated protein alterations:** Several differentially expressed proteins are directly linked to cellular processes essential for membrane integrity, energy metabolism, and bacterial viability, supporting the notion that Mocravimod disrupts key physiological functions required for bacterial survival.

2. **Pathway-level disturbances revealed by enrichment analysis:** GO and KEGG analyses demonstrated significant enrichment of pathways related to metabolism, ribosome function, redox regulation, and lipid-associated processes. These coordinated changes indicate systemic stress and functional imbalance, consistent with a membrane-targeting mode of action.

3. **Correlation with antibacterial phenotypes:** The observed downregulation of virulence-related proteins (e.g., membrane-damaging toxins) and alterations in metabolic and stress-response proteins align well with the experimentally observed growth inhibition, biofilm suppression, and membrane disruption phenotypes. Together, these findings establish a coherent link between protein expression changes, pathway dysfunction, and antibacterial activity.

In addition, to further improve clarity, we have revised the Discussion section to explicitly emphasize the value of proteomic analysis in elucidating the antibacterial mechanism of Mocravimod. We appreciate the reviewer's comment, which has helped us better articulate the mechanistic significance of the proteomic data in the revised manuscript.

Comment R2-6: Line 32: How was the alteration in gene expression measured? Whole-genome sequencing (WGS) alone is not suitable for assessing expression profiles, so clarification of the methodology used for expression analysis is needed.

Response R2-6: We thank the reviewer for this important and technically relevant comment. We agree that whole-genome sequencing (WGS) alone is not suitable for directly assessing gene expression levels, and we appreciate the opportunity to clarify this point.

In this study, WGS was not used to measure alterations in gene expression. Specifically, whole-genome sequencing was performed on both the parental strain *S. aureus* YUSA145 and the KRP-203-induced tolerant strain YUSA145N60. Comparative genomic analysis was then conducted to identify sequence-level differences between the two highly related strains.

Variants including single nucleotide polymorphisms (SNP) and indels were identified using MUMmer (v3.23), as described in the Materials and Methods section. This SNP-based comparison allowed us to pinpoint nonsynonymous mutations that arose during prolonged KRP-203 exposure and that may contribute to altered bacterial physiology or drug tolerance. We thank the reviewer for highlighting this important distinction, which has helped us improve the clarity and accuracy of the manuscript.

Comment R2-7: In the study design, please clarify why the antibacterial activity of Mocravimod was evaluated only against Staphylococcus. What was the rationale for not including E. coli or other Gram-negative microorganisms for comparison?
Response R2-7: We thank the reviewer for this thoughtful and relevant question regarding the scope of bacterial species investigated in this study.

The decision to focus exclusively on *Staphylococcus aureus* was based on both clinical relevance and scientific rationale. *S. aureus* remains one of the most prevalent bacterial pathogens isolated from clinical infections worldwide and is consistently ranked among the leading causes of hospital- and community-acquired infections. In particular, the widespread dissemination of methicillin-resistant *S. aureus* (MRSA) continues to pose a major challenge to current antimicrobial therapy, highlighting the urgent need for new or repurposed antibacterial agents targeting this pathogen.

From a practical and experimental perspective, our group has access to a large, well-characterized collection of clinical *S. aureus* isolates, including both MSSA and MRSA strains, which have been extensively validated and categorized in previous studies. This allowed us to perform a systematic and statistically robust evaluation of the antibacterial and antibiofilm activity of Mocravimod in a clinically meaningful context.

In contrast, Gram-negative bacteria such as *Escherichia coli* possess fundamentally different cell envelope architectures and resistance mechanisms. Given that our mechanistic data suggest Mocravimod primarily exerts its antibacterial activity through interactions with the bacterial cell membrane and membrane-associated pathways, directly comparing Gram-positive and Gram-negative organisms within the same experimental framework could introduce significant biological heterogeneity and obscure mechanistic interpretation.

Importantly, the absence of Gram-negative bacteria in the present study does not

imply a lack of potential activity against these organisms. Rather, this work was designed as a focused, mechanism-oriented investigation centered on *S. aureus*. We agree with the reviewer that evaluating the antibacterial spectrum of Mocravimod against additional bacterial species, including Gram-negative pathogens, is of interest and will be considered in future studies to further define the breadth of its antibacterial potential. We have revised the manuscript to clarify this rationale and to emphasize the focused scope of the current study.

Comment R2-8: Line 37: A clear presentation of the study's key findings, along with its limitations and the need for further research, should be provided in this section.
Response R2-8: We thank the reviewer for this constructive and helpful comment. In response, we have revised the concluding statement of the Abstract to provide a clearer and more balanced summary of the study's key findings. In addition to highlighting the antibacterial and antibiofilm activity of KRP-203 against both MSSA and MRSA, we have now explicitly addressed the study's limitations and emphasized the need for further research, including mechanistic clarification, optimization of antibacterial efficacy, and *in vivo* safety evaluation. We believe this revision improves the clarity and scientific rigor of the Abstract and more accurately reflects the scope and implications of our findings.

Comment R2-9: This section should be revised to provide a clear and logical narrative, beginning with the study's objectives, followed by a detailed, stepwise description of the methodology including the number of isolates and the specific methods employed and concluding with a coherent presentation of the results obtained.
Response R2-9: We thank the reviewer for this insightful comment. In response, we have carefully revised the Abstract to improve its clarity and logical structure. The revised Abstract now explicitly states the study objective at the beginning, followed by a stepwise summary of the experimental approaches used to evaluate the antibacterial and antibiofilm activity of KRP-203 against MSSA and MRSA clinical isolates. We also reorganized the presentation of key findings to reflect a clear progression from phenotypic antibacterial effects to mechanistic insights derived from whole-genome sequencing, quantitative proteomics, and membrane function assays. While maintaining the concise format required for an Abstract, methodological and experimental details have been further elaborated in the Materials and Methods and Results sections. We believe these revisions address the reviewer's concern and substantially improve the overall coherence of the Abstract.

Comment R2-10: In this section, the chemical structure, biological activity, and associated pathways of Mocravimod should be thoroughly detailed, and any potential adverse effects on the host should be clearly discussed.
Response R2-10: We sincerely thank the reviewer for this insightful and constructive comment. We agree that the original manuscript did not provide a sufficiently detailed description of Mocravimod. Accordingly, we have substantially revised the manuscript to address this concern. Specifically, we have expanded the Introduction

section to include a clearer and more comprehensive description of Mocravimod's chemical structure, its biological activity as a sphingosine-1-phosphate receptor (S1PR) modulator, and the associated signaling pathways. In addition, we have supplemented the Discussion section with a dedicated paragraph addressing potential host-related adverse effects and the overall safety profile of Mocravimod. We believe these additions improve the completeness and clarity of the manuscript and adequately address the reviewer's concerns.

Comment R2-11: Line 117, This information should be included in the Abstract section.
Response R2-11: We thank the reviewer for this helpful suggestion. In response, we have relocated the description of the initial compound screening process to the Introduction section, where it can be presented with appropriate context and clarity. Owing to the word limit constraints of the Abstract, we were unable to describe the screening strategy in detail. However, the Abstract has been revised to retain a concise summary of the study rationale and key findings, while the screening methodology is now clearly explained in the Introduction.

Comment R2-12: Line 117: The type of screening approach should be described in detail-specifically, whether it involved in silico analysis, computer-based screening, or a structural bioinformatics-based screening method.
Response R2-12: We thank the reviewer for this valuable suggestion. We would like to clarify that Mocravimod was not selected based on in silico analysis, computer-based screening, or structural bioinformatics approaches, nor on prior reports of antibacterial activity or clinical observations related to bacterial infections. Instead, its antibacterial potential was identified through an experimental phenotypic screening of a focused library of FDA-approved clinical compounds purchased from MedChemExpress (MCE).

During this screening, compounds were directly evaluated for their inhibitory activity against Staphylococcus aureus using standard microbiological assays. To improve clarity, we have explicitly added this information to the Materials and Methods section under "Minimum Inhibitory Concentration (MIC) Determination," specifying the source of the compound library. We hope this revision more clearly describes the screening approach and addresses the reviewer's concern.

Comment R2-13: In this screening, no results were presented for Gram-negative bacteria, particularly E. coli. It should be clarified whether the study intentionally focused only on Staphylococcus and, if so, the rationale for this choice should be explained.
Response R2-13: We thank the reviewer for this thoughtful and relevant question regarding the scope of bacterial species investigated in this study.

The decision to focus exclusively on *Staphylococcus aureus* was based on both

clinical relevance and scientific rationale. *S. aureus* remains one of the most prevalent bacterial pathogens isolated from clinical infections worldwide and is consistently ranked among the leading causes of hospital- and community-acquired infections. In particular, the widespread dissemination of methicillin-resistant *S. aureus* (MRSA) continues to pose a major challenge to current antimicrobial therapy, highlighting the urgent need for new or repurposed antibacterial agents targeting this pathogen.

From a practical and experimental perspective, our group has access to a large, well-characterized collection of clinical *S. aureus* isolates, including both MSSA and MRSA strains, which have been extensively validated and categorized in previous studies. This allowed us to perform a systematic and statistically robust evaluation of the antibacterial and antibiofilm activity of Mocravimod in a clinically meaningful context.

In contrast, Gram-negative bacteria such as *Escherichia coli* possess fundamentally different cell envelope architectures and resistance mechanisms. Given that our mechanistic data suggest Mocravimod primarily exerts its antibacterial activity through interactions with the bacterial cell membrane and membrane-associated pathways, directly comparing Gram-positive and Gram-negative organisms within the same experimental framework could introduce significant biological heterogeneity and obscure mechanistic interpretation.

Importantly, the absence of Gram-negative bacteria in the present study does not imply a lack of potential activity against these organisms. Rather, this work was designed as a focused, mechanism-oriented investigation centered on *S. aureus*. We agree with the reviewer that evaluating the antibacterial spectrum of Mocravimod against additional bacterial species, including Gram-negative pathogens, is of interest and will be considered in future studies to further define the breadth of its antibacterial potential. We have revised the manuscript to clarify this rationale and to emphasize the focused scope of the current study.

Comment R2-14: Line 136, Since the isolates exhibit varying minimum inhibitory concentrations (MICs) for different antibiotics, it is necessary to compare the effect of the tested agent alongside more than one antibiotic, rather than limiting the comparison to vancomycin alone.
Response R2-14: We thank the reviewer for this thoughtful and constructive comment. We fully agree that, from a strictly comparative antimicrobial efficacy perspective, evaluating a novel agent alongside multiple clinically used antibiotics can be informative. However, we would like to clarify the rationale underlying our experimental design and the scope of the present study.

First, we acknowledge that although KRP-203 exhibits clear antibacterial activity against *Staphylococcus aureus*, its MIC values (approximately 12 μg/mL) are higher than those of several first-line clinical antibiotics, such as vancomycin (typically ~2

μg/mL). Therefore, the primary objective of this study was not to position KRP-203 as superior to existing antibiotics in terms of absolute MIC values, but rather to characterize its antibacterial and antibiofilm activity and to elucidate its membrane-targeting mechanism of action as a repurposed compound.

Second, in the time-kill kinetic assays, vancomycin was selected as a comparator because it is a well-established standard-of-care agent for *S. aureus* infections and has extensively validated bactericidal activity in both MSSA and MRSA. The comparison was included specifically to contextualize the bactericidal kinetics of KRP-203, and our results unexpectedly demonstrated more rapid bacterial killing by KRP-203 under the tested conditions. This comparison was therefore intended to highlight this kinetic feature rather than to provide a comprehensive efficacy ranking against multiple antibiotics.

Third, we agree with the reviewer that lowering the effective MIC of KRP-203 is a critical consideration for its future clinical applicability. In this regard, we have explored combination strategies, including a checkerboard assay assessing the interaction between KRP-203 and vancomycin. Although no synergistic effect was observed in this initial analysis, we plan to extend these studies to include combinations with other classes of antibiotics in future work.

In addition, although not included in the current manuscript, we have identified a structural analog of KRP-203 that exhibits markedly enhanced antibacterial activity against *S. aureus*, with MIC values reduced to approximately 3 μg/mL. In the future, we will focus on comparative structure-activity relationship (SAR) analyses between KRP-203 and its analogs, with the aim of optimizing antibacterial potency while retaining the membrane-targeting mechanism.

Taken together, we believe that the current study appropriately focuses on mechanistic characterization and proof-of-concept antibacterial activity. Broader antibiotic comparisons and optimization strategies will be the subject of subsequent investigations.

Comment R2-15: Line 145, Table 1: Did you compare the MIC of KRP-203 against Gram-positive bacterial isolates with its clinically applicable dosage?

Response R2-15: We greatly appreciate the reviewer's critical question, which prompts a more in-depth discussion on the translational relevance of KRP-203 against *S. aureus*. KRP-203 is clinically approved for improving the prognostic treatment of patients with hematological malignancies after hematopoietic stem cell transplantation (HSCT), and we retrieved its clinical pharmacokinetic (PK) data from published studies, which showed that the serum peak concentration (Cmax) under the recommended clinical dosage for its original indication is indeed lower than the *in vitro* MIC of KRP-203 against *S. aureus* observed in our study. However, we argue that this apparent discrepancy does not negate the potential of KRP-203 for S. aureus

infection, based on two key considerations:

1. **Serum Cmax does not represent the drug concentration at the *S. aureus* infection site:** The serum PK parameters of KRP-203 for its original indication reflect the drug concentration in the systemic circulation, but antibacterial efficacy depends primarily on the drug concentration at the local infection site rather than serum concentration. For *S. aureus* infections (e.g., skin and soft tissue infections, osteomyelitis, pneumonia), many drugs exhibit tissue-specific enrichment characteristics that are not reflected in serum concentrations. Unfortunately, the existing clinical PK data of KRP-203 only report serum concentrations, and the tissue distribution profile (especially in common *S. aureus* infection sites) remains unknown. This is a key gap that our follow-up studies will address.

2. **The dosage regimen for the original indication is not optimized for *S. aureus* infection:** The clinical dosage of KRP-203 is designed to achieve therapeutic effects for its original disease, which does not necessarily match the dosage required for antibacterial activity. For repurposed antibacterial drugs, dosage adjustment or administration route optimization is a common and feasible strategy to increase local effective concentrations. (1) Increased administration frequency: Shortening the dosing interval can maintain a higher steady-state concentration at the infection site without exceeding the maximum tolerated dose (MTD) of the drug; (2) Local administration: For localized *S. aureus* infections (e.g., skin abscesses, wound infections), topical application (e.g., ointment, gel) of KRP-203 can directly deliver high concentrations of the drug to the infection site, avoiding the limitations of systemic administration (e.g., hepatic metabolism, renal excretion); (3) Combination therapy: Combining KRP-203 with other antibiotics (e.g., β-lactams, macrolides) can produce synergistic antibacterial effects, thereby reducing the effective MIC of KRP-203 and making it achievable at lower concentrations.

We acknowledge that the lack of tissue concentration data of KRP-203 in *S. aureus* infection models is a limitation of the current study. To address this, we will implement the following plans in future work: (1) Establish animal models of *S. aureus* infection (e.g., mouse skin abscess model, rat osteomyelitis model) and determine the concentration of KRP-203 in serum and infected tissues using LC-MS/MS; (2) Explore optimized dosage regimens (e.g., local administration, increased frequency) to achieve effective concentrations at infection sites while ensuring safety.

In conclusion, although the serum Cmax of KRP-203 under its original clinical dosage is lower than the *in vitro* MIC against *S. aureus*, the discrepancy can be explained by the dissociation between serum and tissue concentrations, as well as the lack of dosage optimization for antibacterial purposes. Our study provides the first evidence of the direct antibacterial activity of KRP-203 against *S. aureus* and its underlying mechanism, laying a foundation for subsequent translational studies.

Comment R2-16: Line 164 : It is necessary to demonstrate the proposed mechanism by which KRP-203 inhibits biofilm formation or activity in the tested bacterial strains.

Response R2-16: We thank the reviewer for this important and constructive comment. In the present study, we demonstrated the antibiofilm activity of KRP-203 primarily through phenotypic assays, showing that KRP-203 significantly inhibited biofilm formation and reduced bacterial viability within mature biofilms. In parallel, multiple independent experiments (membrane depolarization, permeabilization and lipid-binding assays) consistently indicated that KRP-203 exerts a pronounced disruptive effect on the *S. aureus* cell membrane, particularly through interactions with membrane phospholipids.

Based on these findings, we propose that membrane disruption represents a key upstream event underlying the antibiofilm activity of KRP-203. In *Staphylococcus aureus*, the cell membrane and biofilm development are functionally and structurally interconnected. The bacterial membrane plays a central role in (1) initial surface attachment via membrane-anchored adhesins, (2) regulation of biofilm-associated pathways through membrane-localized two-component systems and quorum-sensing networks, and (3) maintenance of biofilm-associated stress tolerance by sustaining membrane potential, efflux activity, and extracellular matrix production. Disruption of membrane integrity is therefore expected to interfere with multiple stages of biofilm formation and stability.

At this stage, while we have experimentally validated the membrane-targeting activity of KRP-203, we have not yet identified a specific downstream biofilm-related pathway or molecular target directly responsible for the observed antibiofilm phenotype. Consistent with this, whole-genome sequencing and quantitative proteomics did not reveal a single, previously established biofilm-specific regulator that could fully account for this effect. We have therefore refrained from over-interpreting the data and present membrane perturbation as a plausible and biologically supported mechanism rather than a fully resolved pathway.

To address this limitation, we have explicitly acknowledged it in the Discussion section and outlined future directions. These include targeted studies such as qRT-PCR analysis of adhesion-related genes (e.g., fnbA, clfA), immunoblotting to assess adhesin expression, and pathway-focused investigations to further dissect how membrane disruption by KRP-203 influences biofilm initiation and maturation. We believe these follow-up studies will help clarify the precise molecular mechanisms linking membrane damage to biofilm inhibition.

Comment R2-17: In need to explain if the result gotten is logical or some of them is unexpected.

Response R2-17: We thank the reviewer for this insightful comment. Overall, the results obtained in this study are logically consistent with our central hypothesis that

KRP-203 exerts antibacterial activity primarily through membrane-associated mechanisms. The observed membrane depolarization, increased permeability, phospholipid-binding properties, and reduced activity upon lipid supplementation collectively support a coherent and biologically plausible mode of action.

At the same time, we acknowledge that certain findings, such as the remarkable bactericidal activity of KRP-203, identification of resistance-associated mutations in glnQ and BCAT, and the downregulation of the virulence factor HlgC, were not initially anticipated. However, these results can be reasonably explained in the context of membrane homeostasis, amino acid metabolism, and stress adaptation, which are increasingly recognized as interconnected processes influencing bacterial survival, biofilm formation, and antibiotic tolerance.

To address the reviewer's concern, we have revised the Discussion section to more explicitly distinguish between expected observations that support our working hypothesis and unexpected findings that provide new mechanistic insights and generate testable hypotheses for future investigation. We believe this clarification improves the interpretability and logical flow of the Discussion.

Comment R2-18: Clearly explain the limitations of the study and recommend further research.

Response R2-18: We thank the reviewer for this valuable suggestion. A dedicated section addressing the limitations of the study and directions for future research was already included in the Discussion. In response to the reviewer's comment, we have revised and expanded this section to improve clarity and completeness, explicitly highlighting the limitations related to in vivo validation, resistance mechanism confirmation, membrane-targeting evidence, and, importantly, the currently unresolved molecular basis of KRP-203-mediated biofilm inhibition. Corresponding future research directions have been clearly outlined for each limitation. We believe these revisions strengthen the Discussion and better contextualize the scope and implications of our findings.

Comment R2-19: Include a section that highlights and explains the clear contributions and implications of this study.

Response R2-19: We thank the reviewer for this insightful suggestion. In response, we have added a dedicated subsection in the Discussion section. This new section explicitly summarizes the key scientific contributions of our work, including the identification of KRP-203 as a repurposed antibacterial and antibiofilm agent against clinical *S. aureus* isolates, the mechanistic insights into its membrane-targeting activity, and the broader implications for antibacterial drug repurposing and optimization strategies. We believe this addition improves the clarity and impact of the Discussion.

Comment R2-20: Line 431: Please provide more details on the source of the bile

samples, including the host species from which they were collected, as well as the methods used for bacterial isolation.

Response R2-20: We thank the reviewer for carefully identifying this issue. Upon thorough re-examination of the clinical isolate records, we confirmed that a small number of *Staphylococcus aureus* isolates in our strain collection were originally obtained from bile samples of patients in the hepatobiliary surgery department. However, we would like to clarify that none of these bile-derived isolates were included in the experiments presented in this study.

To avoid any potential confusion and to ensure the accuracy and clarity of the manuscript, we have therefore removed the description of bile as a sample source from the Materials and Methods section. We appreciate the reviewer for drawing our attention to this oversight, which has helped us improve the precision of the manuscript.

Once again, we sincerely appreciate the constructive feedback from the editor and reviewers. Your thoughtful comments have been invaluable in enhancing the quality of our manuscript. We hope that the revised version now meets the standards of the journal and addresses all concerns raised.

Thank you very much for your time and consideration. We look forward to your favorable response.

Sincerely,

Peiyu Li (on behalf of all authors)
Department of Infectious Diseases and Shenzhen Key Laboratory for Endogenous Infections, Shenzhen Nanshan People's Hospital, Affiliated Nanshan Hospital of Shenzhen University, Shenzhen, 518052, China
Email: peiyuli12@fudan.edu.cn

Re: Spectrum02263-25R1 (**Mocravimod as a repurposing drug against clinical isolates of *Staphylococcus aureus* by targeting cell membrane**)

Dear Dr. Peiyu Li:

Your manuscript has been accepted, and I am forwarding it to the ASM production staff for publication. Your paper will first be checked to make sure all elements meet the technical requirements. ASM staff will contact you if anything needs to be revised before copyediting and production can begin. Otherwise, you will be notified when your proofs are ready to be viewed.

Data Availability: ASM policy requires that data be available to the public upon online posting of the article, so please verify all links to sequence records, if present, and make sure that each number retrieves the full record of the data. If a new accession number is not linked or a link is broken, provide production staff with the correct URL for the record. If the accession numbers for new data are not publicly accessible before the expected online posting of the article, publication may be delayed; please contact ASM production staff immediately with the expected release date.

Publication Fees: For information on publication fees and which article types have charges, please visit our website. We have partnered with Copyright Clearance Center (CCC) to collect author charges. If fees apply to your paper, you will receive a message from no-reply@copyright.com with further instructions. For questions related to paying charges through RightsLink, please contact CCC at ASM_Support@copyright.com or toll free at +1-877-622-5543. CCC makes every attempt to respond to all emails within 24 hours.

ASM Membership: Corresponding authors may join or renew ASM membership to obtain discounts on publication fees. Need to upgrade your membership level? Please contact Customer Service at Service@asmusa.org.

Embargo Policy: A press release may be issued as soon as the manuscript is posted on the Spectrum Latest Articles webpage. The corresponding author will receive an email with the subject line "ASM Journals Author Services Notification" when the article is available online.

The ASM Journals program strives for constant improvement in our submission and publication process. Please tell us how we can improve your experience by taking this quick Author Survey.

Thank you for submitting your paper to Spectrum.

Sincerely,
Kai Zhou
Editor
Microbiology Spectrum

Reviewer #2 (Comments for the Author):

Thank you for your careful revisions.
