# Supplementary material for: Mocravimod as a repurposing drug against clinical isolates of Staphylococcus aureus by targeting cell membrane
Source: Microbiol Spectr. 2026 Jul 27;14(9):e02263-25. doi: 10.1128/spectrum.02263-25 (PMC13532308; doi:10.1128/spectrum.02263-25)
Supplement: Supplementary file 2 [file spectrum.02263-25-s0001.docx]

**Table S1.** Specific MICs values of KRP-203 against various bacterial species

| Classfication | Name | KRP-203 MIC (μM) |
| --- | --- | --- |
| MRSA (no XDR isolates) | CHS655 | 12.5 |
|  | CHS684 | 12.5 |
|  | CHS686 | 50 |
|  | CHS692 | 25 |
|  | CHS736 | 50 |
|  | CHS791 | 25 |
|  | HAMRSA005 | 25 |
|  | HaMRSA022 | 25 |
|  | HaMRSA047 | 25 |
|  | HaMRSA048 | 25 |
|  | HaMRSA057 | 25 |
|  | HaMRSA075 | 50 |
|  | HaMRSA076 | 50 |
|  | YUSA015 | 50 |
|  | YUSA132 | 50 |
|  | YUSA143 | 25 |
|  | YUSA213 | 25 |
|  | YUSA218 | 25 |
|  | YUSA139 | 12.5 |
|  | YUSA145 | 12.5 |
| MSSA | HaMRSA106 | 50 |
|  | HaMRSA109 | 50 |
|  | HAMRSA114 | 25 |
|  | HAMRSA123 | 25 |
|  | YUSA006 | 12.5 |
|  | YUSA009 | 25 |
|  | YUSA013 | 25 |
|  | YUSA021 | 50 |
|  | YUSA025 | 50 |
|  | YUSA050 | 50 |
|  | YUSA098 | 25 |
|  | YUSA100 | 50 |
|  | YUSA111 | 25 |
|  | YUSA134 | 12.5 |
|  | YUSA135 | 12.5 |
|  | YUSA152 | 12.5 |
|  | YUSA169 | 50 |
|  | SA113 | 12.5 |
|  | CHS101 | 12.5 |
| E. *faecium* | EF16M04 | 12.5 |
|  | EF16M06 | 12.5 |
|  | EF16M08 | 12.5 |
|  | EF16M10 | 12.5 |
|  | EF16M12 | 12.5 |
|  | EF16M14 | 12.5 |
|  | EF16M16 | 12.5 |
|  | EF16M20 | 12.5 |
|  | EF16M22 | 12.5 |
|  | EF16M26 | 12.5 |
|  | EF16M28 | 12.5 |
|  | EF16M30 | 12.5 |
|  | EF16M32 | 12.5 |
|  | EF16M34 | 12.5 |
|  | EF16M38 | 12.5 |
|  | EF16M40 | 12.5 |
|  | EF16M42 | 12.5 |
|  | EF16M45 | 12.5 |
|  | EF16M48 | 12.5 |
| E. *faecalis* | Oglrf | 25 |
|  | EF16C067 | 12.5 |
|  | EF16C047 | 25 |
|  | EF16C032 | 25 |
|  | EF16C023 | 25 |
|  | EF16C003 | 25 |
|  | EF16C106 | 25 |
|  | EF16C124 | 25 |
|  | EF16C126 | 25 |
|  | EF16C166 | 25 |
|  | EF16C168 | 25 |
|  | EF16C340 | 25 |
|  | EF16C350 | 25 |
|  | EF16C353 | 25 |
|  | EF16C359 | 25 |
|  | EF16C348 | 25 |
|  | ATCC29212 | 12.5 |
|  | EF16C030 | 25 |
|  | EF16C051 | 6.25 |
|  | EF16C152 | 6.25 |
|  | EF16C396 | 12.5 |
| S. *Epidermids* | 1457 | 12.5 |
|  | HASE12 | 12.5 |
|  | HASE28 | 12.5 |
|  | HASE30 | 12.5 |
|  | HASE32 | 12.5 |
|  | HASE34 | 12.5 |
|  | HASE38 | 12.5 |
|  | HASE42 | 12.5 |
|  | HASE44 | 25 |
|  | HASE45 | 12.5 |
|  | HASE48 | 12.5 |
|  | HASE50 | 25 |
|  | HASE52 | 25 |
|  | HASE54 | 12.5 |
|  | HASE56 | 12.5 |
|  | HASE58 | 12.5 |
|  | HASE60 | 12.5 |
|  | HASE62 | 12.5 |
|  | HASE64 | 12.5 |
|  | HASE66 | 12.5 |
|  | HASE68 | 12.5 |
|  | HASE70 | 25 |
